# Proteomic analysis of human kidney biopsies unveils emerging acute kidney injury very early after liver graft reperfusion

**DOI:** 10.1186/s12967-025-06695-w

**Published:** 2025-06-16

**Authors:** Åsa Norén, Roberto Boi, Rille Pullerits, Johan Mölne, Kerstin Ebefors, Styrbjörn Friman, Carina Sihlbom, Gustaf Herlenius, Jenny Nyström, Mihai Oltean

**Affiliations:** 1 The Transplant Institute, Sahlgrenska University Hospital, 413 45 Gothenburg, Sweden; 2 Institute of Clinical Sciences, Department of Surgery, Sahlgrenska Academy, University of Gothenburg, Gothenburg, Sweden; 3 Institute of Neuroscience and Physiology, Department of Physiology, Sahlgrenska Academy, University of Gothenburg, Gothenburg, Sweden; 4 Institute of Medicine, Department of Rheumatology and Inflammation Research, Sahlgrenska Academy, University of Gothenburg, Gothenburg, Sweden; 5 Department of Clinical Immunology and Transfusion Medicine, Sahlgrenska University Hospital, Gothenburg, Sweden; 6 Clinical Pathology, Sahlgrenska University Hospital, Gothenburg, Sweden; 7 Department of Laboratory Medicine, Institute of Biomedicine, Sahlgrenska Academy, University of Gothenburg, Gothenburg, Sweden; 8 Proteomics Core Facility, Sahlgrenska Academy, University of Gothenburg, Gothenburg, Sweden

## Abstract

**Background:**

Acute kidney injury (AKI) is a frequent complication after liver transplantation (LT) and is associated with morbidity, mortality, and late development of chronic kidney disease. Risk factors for AKI after LT include patient, perioperative and graft-related factors. The exact renal molecular mechanisms behind AKI in LT are unclear.

**Methods:**

Alterations in the proteome were investigated in kidney biopsies from 21 patients undergoing LT using quantitative proteomics. The most upregulated protein was validated using immunohistochemistry. In addition, serum levels of interleukin (IL)-33, insulin-like growth factor binding protein (IGFBP)-7 and high-mobility group box (HMGB)-1 were analyzed. In silico data validation was performed using 14 recently published proteomics and transcriptomics datasets.

**Results:**

In post-reperfusion biopsies, we identified 731 differentially regulated proteins between patients with and without AKI. The most upregulated pathways were related to inflammation, integrin signaling and extracellular matrix (ECM) remodeling. The most downregulated pathways were traceable to a mitochondrial origin. HMGB-1 was found to be already upregulated (15%) 2 h after LT in patients who later developed AKI. The AKI group also showed upregulation of the alarmin IGFBP-7, caspases 1, 4 and 8, nuclear factor kappa B subunits, and the inflammasome adaptor protein PYCARD. Circulating IL-33 and HMGB-1 (but not IGFBP-7) increased during LT but returned to normal levels within 24 h. Altogether, these findings indicate ongoing inflammatory signaling activity in the kidneys of LT recipients who ultimately develop moderate or severe AKI shortly after liver graft reperfusion.

**Conclusions:**

LT induces extensive alarmin signaling and ECM remodeling in the kidneys of recipients who develop postoperative AKI. Further strategies to curtail this phenomenon are mandated.

*Trial registration* https://www.researchweb.org/is/en/vgr/project/278585, Registered 24 May 2022 (Retrospectively registered).

**Supplementary Information:**

The online version contains supplementary material available at 10.1186/s12967-025-06695-w.

## Introduction

Liver transplantation (LT) is the treatment of choice for a wide range of end-stage liver diseases, and one-year patient and graft survival rates currently exceed 90% [1]. However, long-term results are less impressive, as various co-morbidities after transplantation negatively affect survival. Two such conditions that develop after LT, acute kidney injury (AKI) and chronic kidney disease (CKD), have been repeatedly found to be associated with an elevated risk of death [2, 3]. Despite their known negative impacts, the prediction and timely diagnosis of AKI and CKD are difficult; there is a lack of effective biomarkers as their definitions remain heterogeneous [4]. For instance, several studies have indicated that neutrophil gelatinase-associated lipocalin (NGAL) peaks 2 h after liver graft reperfusion [5–7]. However, a meta-analysis based on 11 studies and 563 patients concluded that, while NGAL can predict AKI after liver transplantation, the great variation between cut-off values and definitions of AKI leaves its value uncertain. Several other biomolecules are still being assessed [7]. However, the narrow window of opportunity to administer the few available treatment options and supportive strategies against AKI is frequently missed.

AKI, reflected by a rapid increase in serum creatinine, a decrease in urine output, or both, is frequent following LT. Depending on the criteria used to define AKI, its incidence varies broadly between 10–80% [8–10]. We previously reported a high incidence of AKI within 48 h of LT, despite adequate pretransplant renal function and unremarkable renal histology at the beginning of the transplant procedure [11]. Impaired renal oxygenation in the presence of hyperdynamic systemic circulation and renal vasodilation, has also been described, resulting in early, severe decline of renal function after LT [12]. Additionally, we have recently shown that grafts of LT recipients who develop early AKI have a distinct proteomic profile compared to those showing normal post-transplant renal function, suggesting that the quality of liver graft cold storage may also be a factor in the development of AKI after LT [13].

The etiology of AKI after LT is poorly understood and likely multifactorial. Traditionally, it has been attributed to the hemodynamic instability and subsequent renal ischemia during LT, or the use of nephrotoxic drugs such as calcineurin inhibitors [10, 14]. Much of the current understanding of the pathogenesis of AKI has been extrapolated from in vitro cell studies, animal models, and/or postmortem human observations. To our knowledge, renal biopsies have not been collected during clinical LT. Although valuable information can be obtained experimentally, animal studies carry significant limitations due to differences between species and strains regarding susceptibility, multiple associated interventions, and comorbidities in the clinical setting, as well as mechanisms and site of injury [15] Likewise, postmortem examination is problematic due to autolysis, which results in tubule degenerative changes [16]. Hence, the initial mechanisms and progression of AKI in living humans have been only partially elucidated [15, 17], and studies based on relevant human samples are mandated in order to understand and tackle this major clinical problem.

Converging evidence suggests that early AKI after LT has molecular and subcellular mechanisms rather than structural, morphological causes. Using quantitative proteomics, we set out to identify alteration patterns in kidney biopsies performed during LT. We hypothesized that distinct molecular characteristics, ultimately leading to AKI, could be detectable within minutes of reperfusion of the liver graft.

## Patients and methods

### Patient management

All activities reported herein were consistent with the Declaration of Helsinki (2013), and the study protocol was reviewed and approved by the Regional Ethical Review Board in Gothenburg (Dnr: 598–13). Adult patients undergoing primary, liver-only transplantation using organs from donors after brain death (DBD), and who were able to give a fully informed, written consent, were eligible for inclusion in the trial. Patients were excluded if they needed renal replacement therapy prior to LT.

A detailed description of organ retrieval and the LT procedure is presented elsewhere [11]. In short, following procurement, liver grafts were kept in static cold storage until transplantation. LT was carried out with preservation of the recipient retrohepatic vena cava, without the use of a portocaval shunt or veno-venous bypass. Cold ischemia time (CIT) was defined as the duration from the start of cold perfusion in the donor to portal reperfusion in the recipient. The immunosuppressive regimen consisted of intraoperative induction with intravenous basiliximab (day 0 and postoperative day (POD) 4) and corticosteroids, followed by maintenance immunosuppression based on mycophenolate mofetil (introduced on day 0) and tacrolimus (introduced on POD 3). Patients with primary sclerosing cholangitis or autoimmune hepatitis received additional oral corticosteroids.

### Sampling

During LT, four renal biopsies were obtained from the cranial pole of the right kidney, using a 16-gauge automated iopsy gun. The first two biopsies were performed at the initial phase of the laparotomy and prior to any vascular dissection. The second pair of biopsies was collected at the end of the transplant procedure, approximately 2 h after reperfusion. Biopsies were placed in 4% buffered formalin (one from each time-point) for histologic evaluation, as well as in RNAlater and kept in− 80 °C until proteomic processing. In addition, a liver allograft biopsy was performed 1–2 h after reperfusion to evaluate graft quality.

Blood samples obtained from individuals accepted as living kidney donors served as healthy controls (n = 6).

Blood samples were collected sequentially, before induction of anesthesia, 2–4 h, and 20–24 h after graft reperfusion, respectively. Serum was recovered, spun, and stored at − 80 °C until further analysis.

### Organ injury- definitions and monitoring

Daily liver function tests (aspartate transferase (AST), alanine transaminase (ALT), bilirubin, international normalized ratio (INR)) and serum creatinine levels were used to evaluate hepatic function and recovery or the development of AKI.

The clinical primary endpoint was AKI development within 48 h of graft reperfusion.

The staging of AKI was based on the Kidney Disease: Improving Global Outcome (KDIGO) criteria [18]. The four stages are: AKI stage 0—no AKI; AKI stage 1—rise in serum creatinine of 1.5–1.9 times baseline or an increase of ≥ 26.5 µmol/L within 48 h; AKI stage 2—rise in serum creatinine of 2.0–2.9 times baseline; and AKI stage 3—rise in serum creatinine of 3 times baseline or an increase in serum creatinine over 353.6 µmol/L or need for renal replacement therapy. Patients were considered to have renal dysfunction if they presented with AKI stages 2 or 3. Consequently, LT patients with AKI stages 2 and 3 were collectively called the group with AKI. Patients without any evidence of renal dysfunction (no AKI) formed a control group.

GFR was measured using ^51^chromium EDTA or iohexol at the time of listing for LT.

### Histology

Renal biopsies were fixed in 4% buffered formalin, paraffinized, and cut into five-micron sections. The biopsies were evaluated using slightly modified Banff criteria [19], as previously published [11]. Tubular cells are particularly sensitive to ischemia, and evaluation was mainly focused on them. Using light microscopy, the following parameters were studied: cytoplasmic vacuolization, loss of microvilli (brush border), tubular dilatation, tubular necrosis, nuclear pyknosis, cellular detachment/luminal cells and scored semi-quantitatively according to Goujon JM et al. [20] with some modifications: 0—no abnormalities; 1—lesion affecting < 10% of the kidney sample; 2—lesions affecting 10–50% of samples; and 3 – lesions affecting > 50% of samples.

Immunohistochemistry was used to confirm the results of the global proteomics analysis. In the kidney biopsies obtained after liver graft reperfusion, we studied the expression of matrix metalloproteinase (MMP)−7, which the proteomic analysis identified as the most differentially expressed protein between patients who developed early postoperative AKI and those with an uneventful course. Kidney biopsy sections were deparaffinized and rehydrated, then antigen retrieval was performed by pressure cooking with DIVA decloaker solution (Biocare Medical, Pacheco, CA), followed by dipping in Hot Rinse (Biocare Medical). Sections were then blocked (PBS with 2% FBS, 2% BSA and 0.2% fish gelatin) before adding the primary antibody (MMP-7, Proteintech 67,990–1, Rosemont, IL). The procedure was completed using the Zytochem plus (AP) polymer kit with permanent red kit (Zytomed systems, Berlin, Germany) according to the manufacturer’s instructions. Sections were then counterstained with hematoxylin before dehydration and mounting with Pertex (Histolab Products AB, Västra Frölunda, Sweden). Slides were then examined ‘blindly’ by an experienced pathologist, and protein overall expression was evaluated semi-quantitatively based on the degree of staining, ranging from absent (0), to weakly positive (+), to clearly positive (+ +). The results were then correlated with the presence of AKI (Pearson).

### Global protein quantification

Proteins were extracted and quantified, as described earlier [21]. In short, biopsies were homogenized, and the extracted proteins were digested using the filter-aided sample preparation protocol (FASP). The samples were run in 3 different sets using the two tandem mass tag multiplex TMT 18-plex labeling method (Thermo Fisher Scientific) with respective references. A total of 27 fractions were submitted to offline liquid chromatography analysis, with a 90 min analytical time frame per fraction. Abundant fractions were injected twice. Each TMT set contained 2 to 4 samples per group (AKI stage, biopsy sampling time groups). The mass spectrometry analysis was performed in data-dependent mode (MultiNoch MS3) using an Orbitrap Fusion Lumos Tribrid mass spectrometer (Thermo Fisher Scientific, Waltham, MA).

Protein identification was achieved with Proteome Discoverer (version 2.4.1.15) against the Swissprot *Homo sapiens* database (Jan 2021). The false discovery rate (FDR) was set to 1%. Since an internal reference was present in the three TMT sets, a total peptide amount normalization could be performed with Proteome Discoverer.

### Proteomic analysis

Qlucore Omics Explorer version 3.8 and 3.9 (Qlucore, Lund, Sweden) was used throughout the proteomic analysis. The presence of batch effect was controlled by analyzing the principal component analysis (PCA) plots to highlight separation between the three TMT sets, as well as by evaluating the skewness of the histograms of the variable frequencies (data distribution of all the proteins in each sample). Mean centering normalization of the intensities was used to remove the batch effect [22] between the TMT sets. The resulting normalized ratios were log2 transformed and used for further analysis and data visualization.

Multi-group comparisons were performed using the Limma package in Qlucore [23]. The PCA plots obtained were used to reveal separation between the experimental groups after removal of the batch effect. Missing values were handled as follows. When a single value was missing, group averages were used. If more than 25% of values were missing, the variable (i.e. the protein) was discarded from the analysis.

A paired two-group test was used to highlight differentially regulated proteins in the comparisons before/after transplantation within the same AKI group because the biopsies originated from the kidneys of the same patient before/after liver transplantation. Unpaired tests were used for the comparisons between AKI severity groups (different patients). Differences were considered significant when p < 0.05 and fold change (FC) ± 20%, unless stated otherwise.

### Pathways analysis

Differentially regulated proteins, selected according to the thresholds described in the previous section, were submitted to Reactome for pathway analysis. FDR was set to 5%, and only human pathways were included in the analysis. The lists of proteins were allowed to be matched against disease pathways, except for the ones related to SARS-CoV-2.

For data integration and presentation, we used several online tools. Gene ontology (GO) processes and GO functions were retrieved through the GSEA module in Qlucore, the online database Panther (www.pantherdb.org/), the Genecards database (www.genecards.org/), the ID mapping module in the Uniprot website www.uniprot.org/id-mapping/uniprotkb), and the SynGO online converter tool (www.syngoportal.org/convert).

### In silico* validation*

Validation was performed with Qlucore using 14 published AKI and ischemia reperfusion injury (IRI) proteomic and transcriptomic databases [24–37]. Notably, all the datasets used for the validation applied completely different normalization methods and treatment of missing values, and had different thresholds for the analysis. Therefore, we used the differentially regulated proteins/genes as they appeared in each published dataset. In practice, this means that we used differentially regulated proteins/genes that passed a q < 0.05 significance threshold in the respective data cohorts, but were selected based on different fold change levels.

Pathway analyses were conducted with Reactome and/or Qlucore (GSEA module). Generally, only regulated pathways with q < 0.05 were used for the comparisons. However, because of the lower amounts of identified downregulated proteins and therefore the lower number of matching downregulated pathways, we have included all the pathways passing a less restrictive p < 0.05 significance threshold, thus allowing for higher q values. Data from these less statistically significant pathways have been highlighted in the tables. Information on how to locate the datasets used for validation is contained in Supplementary Table 5.

### Serum analyses

Serum concentrations of three alarmins, interleukin (IL)−33, insulin-like growth factor binding protein (IGFBP)−7, and high-mobility group box (HMGB)−1, were evaluated using an enzyme-linked immunosorbent assay (ELISA). Quantification of IL-33 (BMS2048, ThermoFisher Scientific), IGFBP-7 (EH252RB, ThermoFisher Scientific), and HMGB-1 (ST51011, IBL International, Hamburg, Germany) was performed according to the manufacturers´ protocols. Analytes below the detection limit were input as half the lower limit of detection (LLOD) in order to facilitate statistical analysis.

### Statistical methods

Continuous patient-related variables were expressed as medians with interquartile ranges (IQR) and compared using the Mann–Whitney U-test. Categorical variables were compared using the Chi-squared test or Fisher’s exact test. P-values of less than 0.05 were considered statistically significant. Data were analysed using GraphPad Prism 5.0 (Dotmatics, San Diego, CA).

## Results

### Patient characteristics and clinical outcomes

Between March 2014 and February 2015, 27 nonconsecutive LT patients were enrolled in the study. However, two patients were excluded from the analyses: one patient required early re-transplantation due to primary non function of the graft, and the other had insufficient intra-LT renal biopsy material. In addition, five patients met the KDIGO criteria of AKI 1 and were consequently left out of further analyses. Altogether, 21 patients (eleven with no AKI and ten with AKI) and their intraoperative renal biopsies form the basis of this report. The main donor and recipient characteristics for these 21 LTs are detailed in Table 1.

**Table 1:** Donor, recipient, and perioperative information

	All, n = 21	AKI 0, n = 11	AKI 2/3, n = 10	P-value
Donor				
Age, years	59 (31–65)	45 (25–69)	62 (57–64)	0.65
Gender: female/male	7/14	4/7	3/7	0.56
BMI, kg/m^2^	25 (22–29)	23 (19–25)	29 (24–31)	0.02
Cause of death				
CVA	9	2	7	0.03
Trauma	8	7	1	0.02
Other	4	2	2	1
Duration of ventilation, hours	36 (29–56)	36 (30–65)	38 (27–56)	0.97
DRI	1.74 (1.31–1.88)	1.38 (1.23–1.80)	1.88 (1.58–1.97)	0.01
CIT, min	490 (383–609)	505 (375–596)	455 (386–640)	1
Recipient				
Age, years	49 (34–57)	47 (34–55)	50 (30–63)	0.75
Gender: female/male	6/15	3/8	3/7	0.63
BMI, kg/m^2^	27 (21–31)	26 (19–27)	29 (25–33)	0.078
Diabetes Mellitus	3	1	2	0.59
MELD score	12 (7–16)	9 (7–14)	14 (8–17)	0.13
Ascites	9	3	6	0.39
Etiology of liver disease^a^				
Primary sclerosing cholangitis	8	4	4	1
Alcohol	5	4	1	0.31
HBV	1	1	0	1
HCV	4	1	3	0.31
HCC	7	2	5	0.18
Other^b^	5	2	3	
mGFR, mL/min/1,73 m^2^	101 (92–109)	103 (84–113)	101 (93–103)	0.62
Serum creatinine at admission, μmol/L	65 (56–88)	65 (58–88)	66 (55–93)	0.94
Duration of surgery, hours	6 (6–8)	6 (5.5–7.5)	7 (6–8)	0.37
Intraoperative bleeding, mL	1800 (600–3300)	1000 (500–4500)	1900 (1000–2600)	0.67
Post-reperfusion syndrome	0	0	0	
ICU stay, hours	24 (15.5–56.5)	19 (16–54)	35.5 (15–92.5)	0.48
RRT	2	0	2	
Peak serum AST (within 72 h), U/L	28 (16–77)	23 (16–33)	77 (15–113)	0.14

Data are given as n (%) or median (IQR)

*AKI* acute kideny injury, *AST* aspartate amino transpherase, *BMI* Body mass index, *CIT* cold ischemia time, *CVA* cerebrovascular accident, *DRI* donor risk index, *mGFR* measured glomerular filtration rate, *HBV* hepatitis B virus, *HCC* hepatocellular carcinoma, *HCV* hepatitis C virus, *ICU* intensive care unit, *MELD* Model for End-stage Liver Disease, *RRT* renal replacement therapy

^a^Etiologies are not mutually exclusive

^b^Includes Autoimmune hepatitis (n = 1), Primary biliary chirrosis (n = 1), Hepatitis B virus (n = 1), Polycystic liver disease (n = 1), Familial amyloid polyneuropathy (n = 1), Non-alcoholic fatty liver disease (n = 1)

**Fig. 1:**
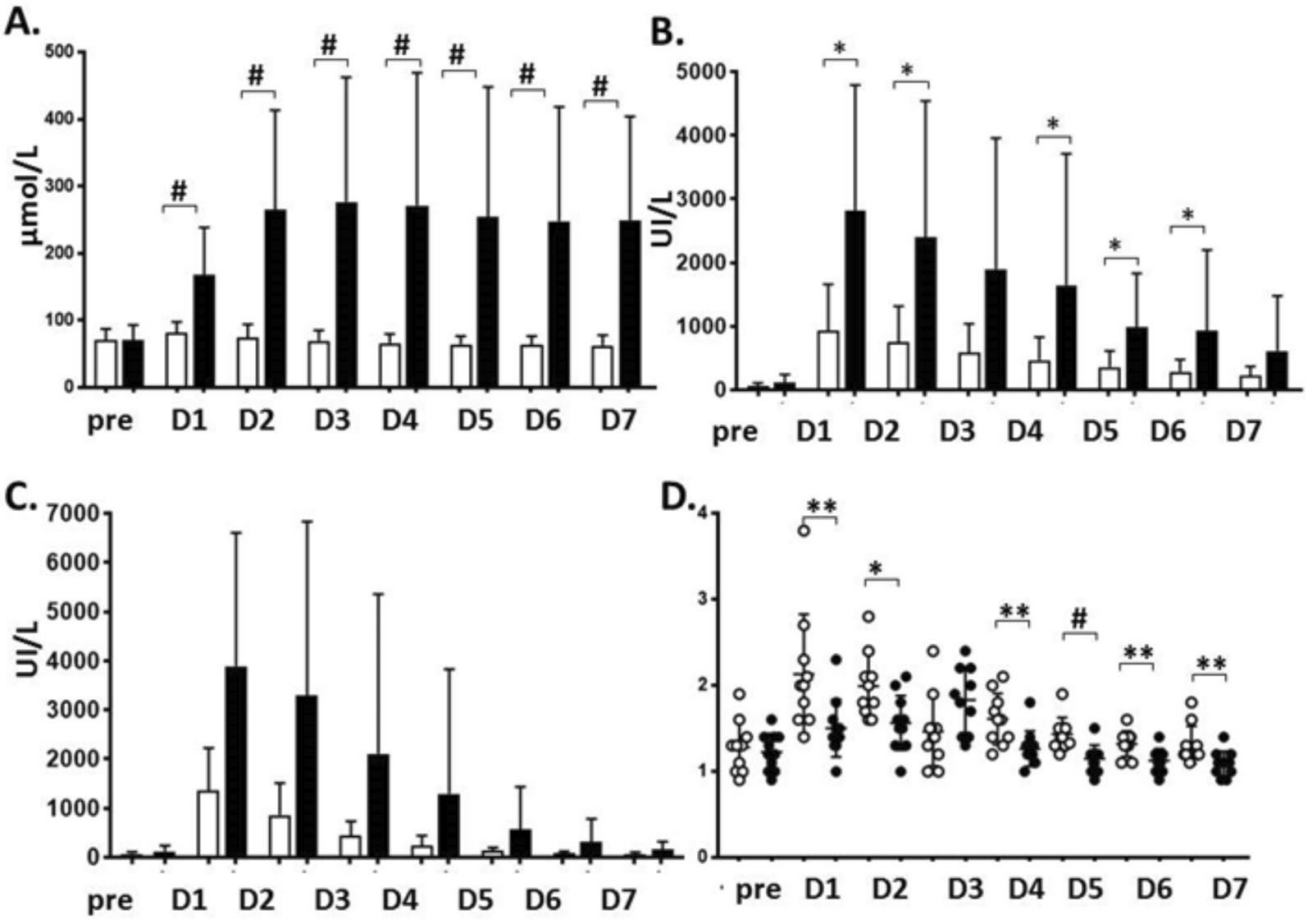
Serum creatinine **A**, Alanine aminotransferase, ALT **B**, Aspartate aminotransferase, AST **C**, and international normalized ratio, INR (**D**) preoperatively and daily during the first week after liver transplantation in patients with postoperative acute kidney injury (AKI, black bars) and no AKI (white bars). * p < 0.5, ** p < 0.01, # p < 0.001

Patients presented with an overall good pre-LT renal function (101 (92–109) ml/min/1.73 m^2^), with no difference between patients who developed AKI 2–3 and those with no AKI. Post-LT creatinine alterations are presented in Fig. 1A. LT resulted in altered liver function tests peaking at POD 1, as shown in Fig. 1 B-D. Patients who developed AKI had poorer liver function tests at corresponding time-points compared to those with no AKI.

Cerebrovascular accident was the main cause of death among donors in the AKI group, whereas trauma was the leading cause of donor death in recipients with no AKI. Notably, donor body mass index (BMI) was significantly higher in patients who developed early AKI compared to others (29 (24–31) and 23 (19–25) kg/m^2^, p = 0.02).

### Renal histology reveals minor differences between kidney biopsies before and after LT

There were only minor differences between biopsy 1 (beginning of surgery) and biopsy 2 (end of surgery). The only feature differing between the time-points was the presence of cytoplasmic vacuoles. Other parameters, such as loss of microvilli/brush border, tubular dilatation or cellular detachment/luminal showed no differences. Further, tubular necrosis and nuclear pyknosis were not observed. Lastly, we found no differences in cytoplasmic vacuolization between the groups with AKI and no AKI in biopsy 2.

### Proteomic analysis reveals quantitative differences after liver reperfusion in the kidneys with and without AKI

**Fig. 2:**
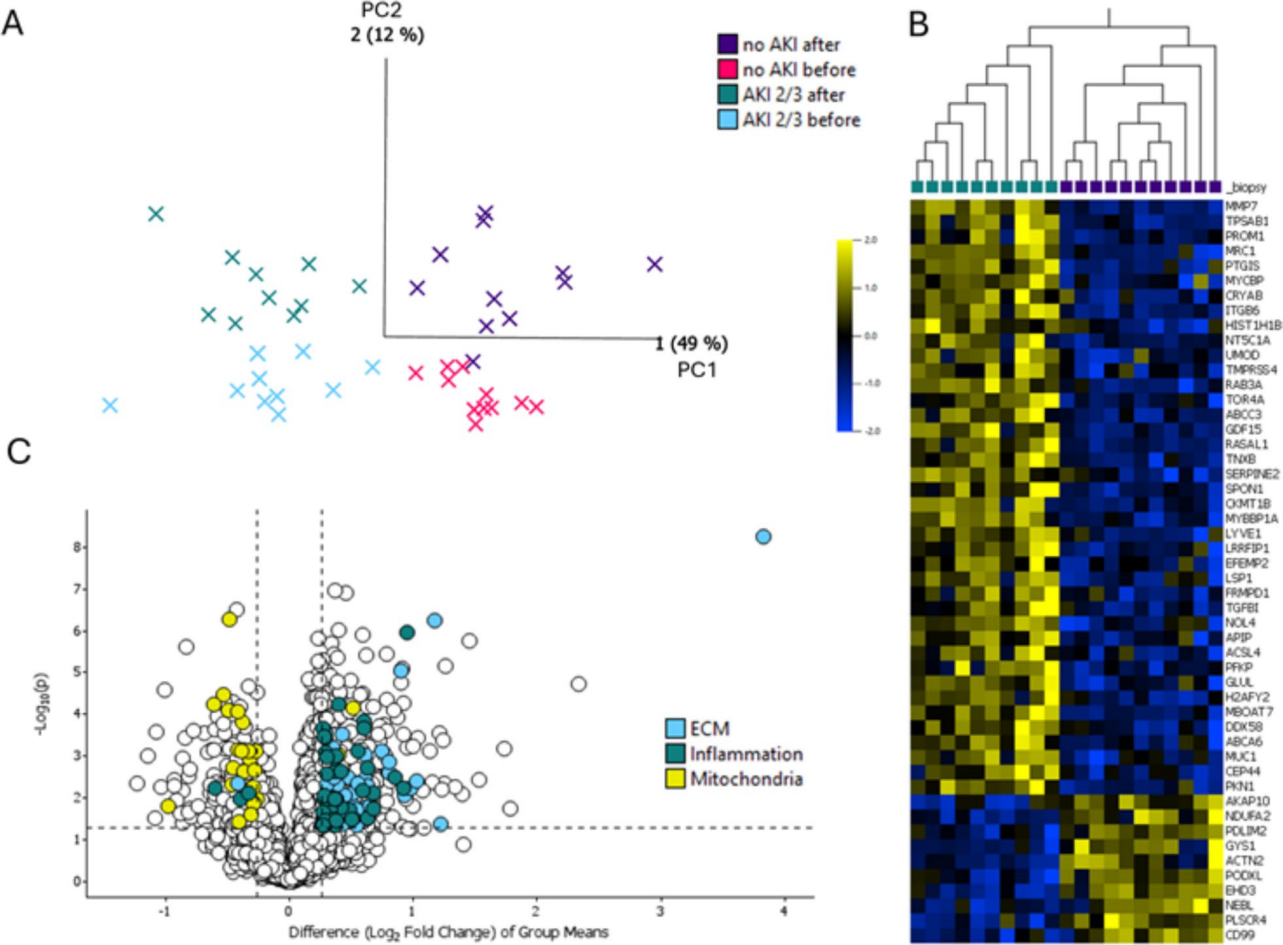
Overview of proteomics results. Principal component (PC) analysis, plotting PC 1 and PC 2, showed clear clustering between the groups with AKI and no AKI. The group with no AKI could not be efficiently clustered “after vs before transplantation”, while a clearer separation existed in the AKI group. **A**. A volcano plot is used to illustrate the variation between the two groups after reperfusion. The dotted lines represent the p-value threshold of 0.05 and the fold change threshold of ± 20% **B**. A selection of the top 50 differentially regulated proteins in the comparison between AKI vs no AKI after reperfusion, ranked by increasing q value, allowed us to obtain a clear hierarchical clustering of the two groups (**C**)

The main focus of this study was to analyse the differences between the kidney biopsies obtained after liver graft reperfusion in patients who developed AKI and in those with an uneventful course. Baseline kidney biopsies obtained at the start of the LT procedure were also processed and analysed. Data-dependent mode analysis of kidney biopsies identified 8144 proteins, of which 6620 were quantified. The PCA method can be used to reduce multidimensional data (n = 6620, i.e. all quantified proteins) to a limited number of coordinates representative of each sample that can be plotted as a bidimensional graph. PCA plots were used to estimate the degree of separation or association between samples belonging to different groups. Each principal component (PC) represents a linear combination of the observed variables – in our case, the quantified proteins. PCA plots showed a clear separation between the group of patients without renal impairment (no AKI) and those with AKI 2/3, regardless of the timing of the biopsy (Fig. 2A). The separation was not perfect in the no AKI group before/after the transplantation.

The comparative analysis of the kidney biopsies obtained after graft reperfusion identified 731 differentially regulated proteins (542 upregulated and 289 downregulated). The thresholds used were p < 0.05 and fold change (FC) ± 20%. The most upregulated protein was MMP-7, while the phospholipid scramblase (PLS) CR4 was the most downregulated one. A complete list is reported in Supplemental Table 1.

The top 50 most differentially regulated proteins in the comparison are reported as a heatmap in Fig. 2B. A clear separation between the groups with AKI 2/3 and no AKI is illustrated by the hierarchical clustering on top of the heatmap. The 100 most up- and downregulated proteins (p < 0.05, ranked by FC) are listed in Table 2.

**Table 2:** Top 100 most up-, and downregulated proteins in the AKI 2/3 vs no AKI groups, comparison after transplantation

UPREG					
Symbol	Name	P-value	FC	Biological function	Biological process
MMP7	Matrix metallopeptidase 7	5.59E-09	14.17	Metalloprotease	Extracellular matrix organization
TPSAB1	Tryptase alpha/beta 1	1.91E-05	5.05	Serine protease	Extracellular matrix disassembly
FAM21C	Family With Sequence Similarity 21 Member C	1.73E-02	3.45	Chaperone	Endosomal transport
PROM1	Prominin 1	6.68E-04	3.32	Actin or actin-binding cytoskeletal protein	Podocyte differentiation
MUC13	Mucin 13, cell surface associated	3.72E-03	2.89	Defense/immunity protein	Maintenance of gastrointestinal epithelium
MRC1	Mannose receptor C-type 1	1.75E-06	2.73	Membrane trafficking regulatory protein	Cellular response to interleukin-4
IGJ	Immunoglobulin lambda constant 7	8.12E-03	2.63	Immunoglobulin	Immune response
SFT2D3	SFT2 domain containing 3	5.64E-03	2.52	Phosphatase	Vesicle-mediated transport
PTGIS	Prostaglandin I2 synthase	6.84E-06	2.39	Prostacyclin synthase	Prostaglandin biosynthetic process
ADH4	Alcohol dehydrogenase 7 (class IV), mu or sigma polypeptide	4.55E-03	2.38	Dehydrogenase	Alcohol metabolic process
MYCBP	MYC binding protein	4.29E-04	2.36	Transcription cofactor	Chromatin remodeling
SLC39A9	Solute carrier family 39 member 9	6.22E-03	2.35	Secondary carrier transporter	Cellular zinc ion homeostasis
COL6A6	Collagen type VI alpha 6 chain	4.35E-02	2.33	Extracellular matrix structural protein	Extracellular matrix organization
CRYAB	Crystallin alpha B	2.72E-04	2.32	Metalloprotease	Microtubule polymerization or depolymerization
ITGB6	Integrin subunit beta 6	5.66E-07	2.25	Integrin	Cell adhesion mediated by integrin
HIST1H1D	H1.5 linker histone, cluster member	4.07E-03	2.22	Histone component	Chromosome condensation
HIST1H1B	H1.3 linker histone, cluster member	7.36E-04	2.19	Histone component	Chromatin organization
NQO1	NAD(P)H quinone dehydrogenase 1	6.46E-03	2.10	Oxidoreductase	Ubiquinone metabolic process
NT5C1A	5′-nucleotidase, cytosolic IA	3.60E-04	2.05	Nucleotidase	Nucleoside metabolic process
SPP1	Secreted phosphoprotein 1	4.05E-03	2.04	Cytokine	Cell adhesion
AKR1B10	Aldo–keto reductase family 1 member B10	1.58E-02	2.00	Reductase	Cellular detoxification of aldehyde
UMOD	Uromodulin	5.78E-04	2.00	Transmembrane signal receptor	Glomerular filtration
COL14A1	Collagen type XIV alpha 1 chain	5.92E-03	1.97	Extracellular matrix structural protein	Extracellular matrix organization
TMPRSS4	Transmembrane serine protease 4	6.07E-04	1.93	Serine protease	Negative regulation of growth rate
RAB3A	RAB3A, member RAS oncogene family	1.09E-06	1.93	GTPase-activating protein	Calcium-ion regulated exocytosis
IGLC7	Immunoglobulin Lambda Constant 7	1.08E-02	1.92	Immunoglobulin	Immune response
CPA3	carboxypeptidase A4	7.31E-03	1.91	Metalloprotease	Angiotensin maturation
CMA1	Chymase 1	8.65E-03	1.90	Serine protease	Angiotensin maturation
TOR4A	Torsin family 4 member A	3.07E-04	1.90	Chaperone	Chaperone-mediated protein folding
ABCC3	ATP binding cassette subfamily C member 3	5.33E-05	1.89	ATP-binding cassette	Transmembrane transport
CES1	Carboxylesterase 1	5.81E-03	1.89	Esterase	Cholesterol biosynthetic process
GDF15	Growth differentiation factor 15	8.01E-06	1.88	Growth factor	transforming growth factor beta receptor signaling pathway
ADH1A	Alcohol dehydrogenase 1 A (class I), alpha polypeptide	4.74E-02	1.88	Dehydrogenase	Alcohol metabolic process
RASAL1	RAS protein activator like 1	1.79E-05	1.87	Protease inhibitor	Cellular response to calcium ion
TNXB	Tenascin XB	9.30E-06	1.87	Extracellular matrix protein	Actin cytoskeleton organization
SERPINE2	Serpin family E member 2	1.19E-04	1.83	Transmembrane signal receptor	Regulation of cell migration
C9	Complement Component C9	3.19E-03	1.81	Complement component	Complement activation
SPON1	Spondin 1	7.99E-05	1.79	Cell adhesion molecule	Cell adhesion
SFRP1	Secreted frizzled related protein 1	2.88E-02	1.78	Frizzled protein	Canonical Wnt signaling pathway
SLC5A8	Solute carrier family 5 member 8	3.95E-03	1.76	Secondary carrier transporter	Ion transport
LTBP2	Latent transforming growth factor beta binding protein 3	1.34E-03	1.75	Extracellular matrix structural protein	Transforming growth factor beta receptor signaling pathway
FBLN5	Fibulin 5	2.46E-03	1.74	Scaffold/adaptor protein	Cell–matrix adhesion
MRC2	Mannose receptor C type 2	2.50E-03	1.74	Membrane trafficking regulatory protein	Cellular response to interleukin-4
CKMT1A	Creatine kinase, mitochondrial 1A	4.32E-05	1.72	Amino acid kinase	Phosphocreatine biosynthetic process
PDLIM3	PDZ and LIM domain 3	4.88E-02	1.72	Actin or actin-binding cytoskeletal protein	Actin cytoskeleton organization
MYBBP1A	MYB binding protein 1a	3.70E-06	1.72	Chromatin/chromatin-binding, or -regulatory protein	Chromatin remodeling
VKORC1	Vitamin K epoxide reductase complex subunit 1	1.18E-02	1.72	Oxidoreductase	Xenobiotic metabolic process
LYVE1	Lymphatic vessel endothelial hyaluronan receptor 1	2.54E-04	1.72	Transmembrane signal receptor	Cell–matrix adhesion
UCHL1	Ubiquitin C-terminal hydrolase L1	9.96E-04	1.72	Cysteine protease	Protein deubiquitination
FERMT1	FERM domain containing kindlin 1	7.03E-03	1.71	Extracellular matrix structural protein	Cell–matrix adhesion
DOWNREG					
CALML3	Calmodulin like 3	4.62E-03	0.43	Calmodulin-related	Calcium ion binding
DHRS4L2	Dehydrogenase/reductase 4 like 2	9.88E-04	0.45	Dehydrogenase	Carbonyl reductase
KRT9	Keratin 9	3.04E-02	0.47	Structural protein	Structural constituent of cytoskeleton
CD99	CD99 molecule (Xg blood group)	2.61E-04	0.48	Protein binding	Cell adhesion
FTH1	Ferritin heavy chain 1	5.33E-03	0.49	Storage protein	Ferric iron binding
PLSCR4	Phospholipid scramblase 4	2.58E-05	0.50	Transporter	Phospholipid scramblase activity
CREBBP	CREB binding protein	1.52E-02	0.51	Histone modifying enzyme	Chromatin binding
YIPF5	Yip1 domain family member 5	5.21E-03	0.53	Structural protein	Phospholipid scramblase activity
CIRBP	Cold inducible RNA binding protein	3.75E-03	0.53	RNA metabolism protein	Translation repressor activity
CPNE6	Copine 6	1.13E-02	0.53	Calcium-binding protein	Calcium ion binding
KRT1	Keratin 1	2.33E-02	0.54	Structural protein	Structural constituent of cytoskeleton
ARSF	Arylsulfatase F	6.46E-03	0.55	Hydrolase	Arylsulfatase activity
PTPRO	Protein tyrosine phosphatase receptor type U	1.14E-03	0.55	Transmembrane signal receptor	Wnt-protein binding
DPYD	Dihydropyrimidine dehydrogenase	4.07E-02	0.55	Dehydrogenase	Dihydropyrimidine dehydrogenase
NEBL	Nebulette	3.56E-04	0.56	Actin binding	Focal adhesion
EHD3	EH domain containing 3	2.40E-06	0.56	Membrane traffic protein	Calcium ion binding
DPEP1	Dipeptidase 1	2.97E-03	0.57	Protease	Dipeptidase activity
KRT6B	Keratin 6B	2.24E-02	0.57	Structural protein	Structural constituent of cytoskeleton
TBC1D2B	TBC1 domain family member 2B	1.98E-03	0.57	GTPase-activating protein	GTPase activator activity
DDN	Dendrin	1.80E-02	0.58	Protein binding	Protein binding
FTL	Ferritin light chain	2.90E-02	0.58	Storage protein	Ferric iron binding
PODXL	Podocalyxin like	2.53E-04	0.59	Cell adhesion molecule	Cell adhesion
FGF1	Fibroblast growth factor 1	2.53E-02	0.60	Growth factor	Fibroblast growth factor receptor binding
RBP4	RNA polymerase II subunit D	1.59E-02	0.60	transfer/carrier protein	Retinol transmembrane transporter activity
DSC1	Desmocollin-1	3.68E-02	0.61	Cadherin	Calcium-dependent protein serine/threonine phosphatase regulator activity
ERP27	Endoplasmic reticulum protein 27	2.72E-02	0.61	Chaperone	Chaperone
ABP1	Amine oxidase copper containing 1	2.25E-02	0.62	Oxidase	Calcium ion binding
IGKV2-30	Immunoglobulin kappa variable 2–30	3.28E-02	0.62	Immunoglobulin	Antigen binding
ACTN2	Actinin alpha 2	2.74E-04	0.62	Actin or actin-binding cytoskeletal protein	Actin filament binding
CLEC18A	C-type lectin domain family 18 member A	4.35E-02	0.63	Defense/immunity protein	Polysaccharide binding
GSTA1	Glutathione S-transferase alpha 1	2.53E-02	0.63	Transferase	Fatty acid binding
NPHS2	NPHS2 stomatin family member, podocin	7.88E-04	0.63	Cytoskeletal protein	Actin binding
C1S	Complement C1s	1.74E-02	0.64	Serine protease	Serine-type endopeptidase activity
VMP1	Vacuole membrane protein 1	4.89E-03	0.64	Transmembrane signal receptor	Phospholipid scramblase activity
APOC3	Apolipoprotein C3	2.76E-02	0.64	Apolipoprotein	Phospholipid binding
EMCN	Endomucin	7.79E-03	0.64	Protein binding	Protein binding
GYS1	Glycogen synthase 1	7.11E-04	0.65	Glycogen synthase	Glycogen synthase activity
CLDN2	Claudin 2	2.74E-03	0.65	Tight junction	Identical protein binding
GCDH	Glutaryl-CoA dehydrogenase	3.69E-02	0.65	Dehydrogenase	Fatty-acyl-CoA binding
PDLIM2	PDZ and LIM domain 2	6.86E-04	0.65	Actin or actin-binding cytoskeletal protein	Actin binding
NDUFA2	NADH:ubiquinone oxidoreductase subunit A2	5.88E-05	0.66	Oxidoreductase	NADH dehydrogenase ubiquinone) activity
CDH13	Cadherin 13	4.75E-02	0.66	Cadherin	Calcium ion binding
SLC34A3	Solute carrier family 34 member 3	8.20E-03	0.66	Secondary carrier transporter	Sodium:phosphate symporter activity
AKAP10	A-kinase anchoring protein 10	2.37E-04	0.66	Scaffold/adaptor protein	Protein kinase A binding
MAGI2	Membrane associated guanylate kinase, WW and PDZ domain containing 2	1.09E-02	0.66	Kinase activity	Beta-1 adrenergic receptor binding
TUBB4B	Tubulin beta 4B class IVb	5.93E-03	0.66	Tubulin	Structural constituent of cytoskeleton
CLIC5	Chloride intracellular channel 5	4.11E-03	0.66	Ion channel	Chloride channel activity
THTPA	Thiamine triphosphatase	1.15E-04	0.67	Phosphatase	Hydrolase activity
ALPL	Alkaline phosphatase, biomineralization associated	7.57E-03	0.67	Phosphatase	Alkaline phosphatase activity
IGHV3-74	Immunoglobulin heavy variable 3–74	4.15E-02	0.68	Immunoglobulin	Antigen binding

*FC* fold change

The analysis of baseline kidney biopsies (i.e. at the start of LT) identified 249 differentially regulated proteins (155 upregulated and 94 downregulated, p < 0.05, FC ± 20%, data not shown) in the comparison between the groups with AKI 2/3 and no AKI. The 100 most up- and downregulated proteins for this comparison are reported in Supplementary Table 2.

### Pathway analysis reveals upregulation of inflammation and extracellular matrix (ECM) remodeling, alongside downregulation of mitochondria-related pathways

Pathway analysis was performed using the regulated proteins found using p < 0.05 and FC ± 20% in the group of patients with AKI 2/3 vs no AKI (after LT). The most upregulated pathways after graft reperfusion were related to immunity, inflammation, integrins and ECM signaling. Remarkably, among the most downregulated pathways, many were traceable to a mitochondrial origin (Fig. 2C, Table 3). In Fig. 2C, a volcano plot is used to illustrate the quantitative differences between the proteomes of the AKI 2/3 and no AKI groups after LT reperfusion. Proteins from ECM, inflammation (upregulation) or mitochondrial (downregulation) related pathways are highlighted to visually report the broad changes happening to these pathways.

**Table 3:** Most regulated pathways in the AKI 2/3 vs no AKI groups, comparison after transplantation

Pathway name	Proteins found	Total proteins	P-value	FDR	Direction of regulation
Antigen Presentation: Folding, assembly and peptide loading of class I MHC	26	108	3.91E−12	5.43E− 09	↑
Extracellular matrix organization	44	328	6.28E−11	4.35E−08	↑
Endosomal/Vacuolar pathway	21	82	1.53E−10	7.06E−08	↑
Interferon alpha/beta signaling	31	190	5.11E−10	1.77E−07	↑
Collagen degradation	18	69	2.33E−09	5.39E−07	↑
Interferon Signaling	45	397	6.54E−09	1.29E−06	↑
Degradation of the extracellular matrix	25	148	1.25E−08	2.16E−06	↑
Assembly of collagen fibrils and other multimeric structures	16	67	5.91E−08	8.15E−06	↑
Signaling by PDGF	16	70	1.06E−07	1.22E−05	↑
Antigen processing-Cross presentation	26	195	5.93E−07	5.87E−05	↑
Collagen chain trimerization	12	44	6.77E−07	6.22E−05	↑
ER−Phagosome pathway	24	173	8.11E−07	6.98E−05	↑
Molecules associated with elastic fibres	11	37	8.62E−07	6.98E−05	↑
Integrin cell surface interactions	16	86	1.53E−06	1.18E−04	↑
Terminal pathway of complement	6	8	1.62E−06	1.19E−04	↑
Interferon gamma signaling	29	252	2.45E−06	1.69E−04	↑
Collagen formation	17	104	4.01E−06	2.65E−04	↑
Elastic fibre formation	11	45	5.47E−06	3.45E−04	↑
Collagen biosynthesis and modifying enzymes	13	76	3.38E−05	2.03E−03	↑
Cytokine Signaling in Immune system	74	1115	1.09E−04	6.20E−03	↑
Class I MHC mediated antigen processing & presentation	39	479	1.31E−04	7.22E−03	↑
NCAM1 interactions	9	44	1.47E−04	7.77E−03	↑
ECM proteoglycans	12	79	1.99E−04	1.02E−02	↑
Apoptotic execution phase	9	54	6.38E−04	3.13E−02	↑
Immunoregulatory interactions between a Lymphoid and a non−Lymphoid cell	27	316	6.85E−04	3.22E−02	↑
Activation of Matrix Metalloproteinases	7	35	8.94E−04	4.11E−02	↑
The citric acid (TCA) cycle and respiratory electron transport	19	238	1.76E−09	7.03E−07	↓
Respiratory electron transport	14	118	2.03E−09	7.03E−07	↓
Respiratory electron transport, ATP synthesis, heat production by uncoupling proteins	15	153	6.72E−09	1.55E−06	↓
Complex I biogenesis	8	59	2.36E−06	4.08E−04	↓
Iron uptake and transport	8	83	2.72E−05	3.75E−03	↓
Trans-Golgi Network Vesicle Budding	7	80	1.57E−04	1.80E−02	↓
Lysosome Vesicle Biogenesis	5	43	3.90E−04	3.86E−02	↓
Nephrin family interactions	4	25	4.66E−04	3.93E−02	↓
Mitochondrial biogenesis	8	128	5.10E−04	3.93E−02	↓

FDR: false discovery rate

Regarding the comparison at baseline (AKI 2/3 vs no AKI, before LT), the pathway analysis showed upregulation of early inflammatory signaling and antigen presentation, and downregulation of electron transport and energy metabolism (Supplementary Table 3). During the short time interval between baseline biopsy and post-reperfusion biopsy, proteins involved in ribosomal processes, immunoglobulin regulation and collagen turnover were particularly altered in the AKI 2/3 group compared to the no AKI group (Supplementary Table 4).

In addition to Reactome pathway analysis, we performed a manual search for proteins potentially involved in the transduction of circulating inflammatory signals originating from the liver. We specifically searched for alarmins, inflammasomes, and proteins related to the following pathways: MyD88 (myeloid differentiation primary response), NOD (nucleotide-binding and oligomerization domain), NOD-like receptor (NLR), and toll-like receptor signaling cascades, TRAF (tumor necrosis factor receptor associated factors) induction of interferon, and WNT (wingless-type MMTV integration site family) ligands biogenesis and trafficking (Table 4). Among the identified proteins, HMGB-1 was found to be already upregulated (+ 15%, p = 0.009) 2 h after LT in the patient group that later developed AKI. HMGB-1 is a key protein in the pathogenesis of liver injury and a marker for AKI. Another alarmin, IGFBP-7, was also upregulated (FC + 20%) in the AKI group. Furthermore, there was upregulation of several caspases (CASP1, CASP4, CASP8), nuclear factor kappa B subunits (NFKB, RELA), and the inflammasome adaptor protein PYCARD (PYRIN-PAAD-DAPIN Caspase-Recruitment Domain). All these findings point to ongoing inflammatory signaling activity in the kidneys of LT recipients who ultimately develop moderate or severe AKI, beginning only minutes after liver graft reperfusion.

**Table 4:** Alarmins and proteins from related pathways in AKI 2/3 vs no AKI groups, comparison after transplantation

PROTEIN	Name	P−value	Fold change	Alarmins	Inflammasomes	MyD88 cascade	NLR signaling	NOD 1–2 signaling	Regulation of TLR by endogenous ligand	Toll-like receptor cascades	TRAF6 mediated induction of interferonA-B via NFkb	Trafficking and processing of endosomal TLR	WNT ligand biogenesis and trafficking
AAMP	Angio-associated migratory cell protein	2.11E−03	1.14				x	x					
AKT2	RAC-beta serine/threonine-protein kinase	9.91E−04	1.18										
CASP1	Caspase 1	2.25E−03	1.29		x		x						
CASP8	Caspase 8	1.16E−02	1.22			x	x			x			
CD14	Monocyte differentiation antigen CD14	1.14E−03	1.29			x			x	x			
CD163	Scavenger receptor cysteine-rich type 1 protein M130	3.30E−02	1.42	x									
CUL1	Cullin-1	3.89E−03	1.08			x				x			
DDX58 (RIG-I)	Antiviral innate immune response receptor RIG-I	1.43E−04	1.52								x		
HMGB1	High mobility group protein B1	9.36E−03	1.15	x		x			x	x	x		
HSP90AB1	Heat shock protein HSP 90-beta	2.89E−03	1.15				x						
IGFBP7	Insulin-like growth factor−binding protein 7	1.54E−02	1.23	x	x								
IKBKB	Inhibitor of nuclear factor kappa-B kinase subunit beta	3.20E−03	0.84			x	x			x			
ITGA3	Integrin A3	2.81E−02	0.80	x									
MAP2K1	Mitogen-activated protein kinase kinase 1	1.95E−02	1.26			x				x			
NFKB1	Nuclear factor kappa B subunit 1	4.97E−03	1.14		x	x				x	x		
PPP2R1A	PPP2R2A-interacting phosphatase regulator 1	1.93E−02	1.06			x				x			
PYCARD	PYD and CARD domain containing	6.78E−03	1.51		x		x						
RELA	RELA proto-oncogene, NF-kB subunit	5.35E−02	1.14								x		
SBF1 (MTMR5)	Myotubularin-related protein 5	3.40E−02	1.22										
SUGT1	Protein SGT1 homolog	4.32E−03	1.08		x								
TMED5	Transmembrane emp24 domain-containing protein 5	3.41E−02	1.11										x
TRAF2	TRAF family member associated NFKB activator	3.44E−02	1.30			x				x	x		
UBE2N	Ubiquitin-conjugating enzyme E2 N	4.99E−02	1.08			x	x			x			

### MMP-7 immunostaining confirms differential ECM remodeling in kidneys with and without AKI

**Fig. 3:**
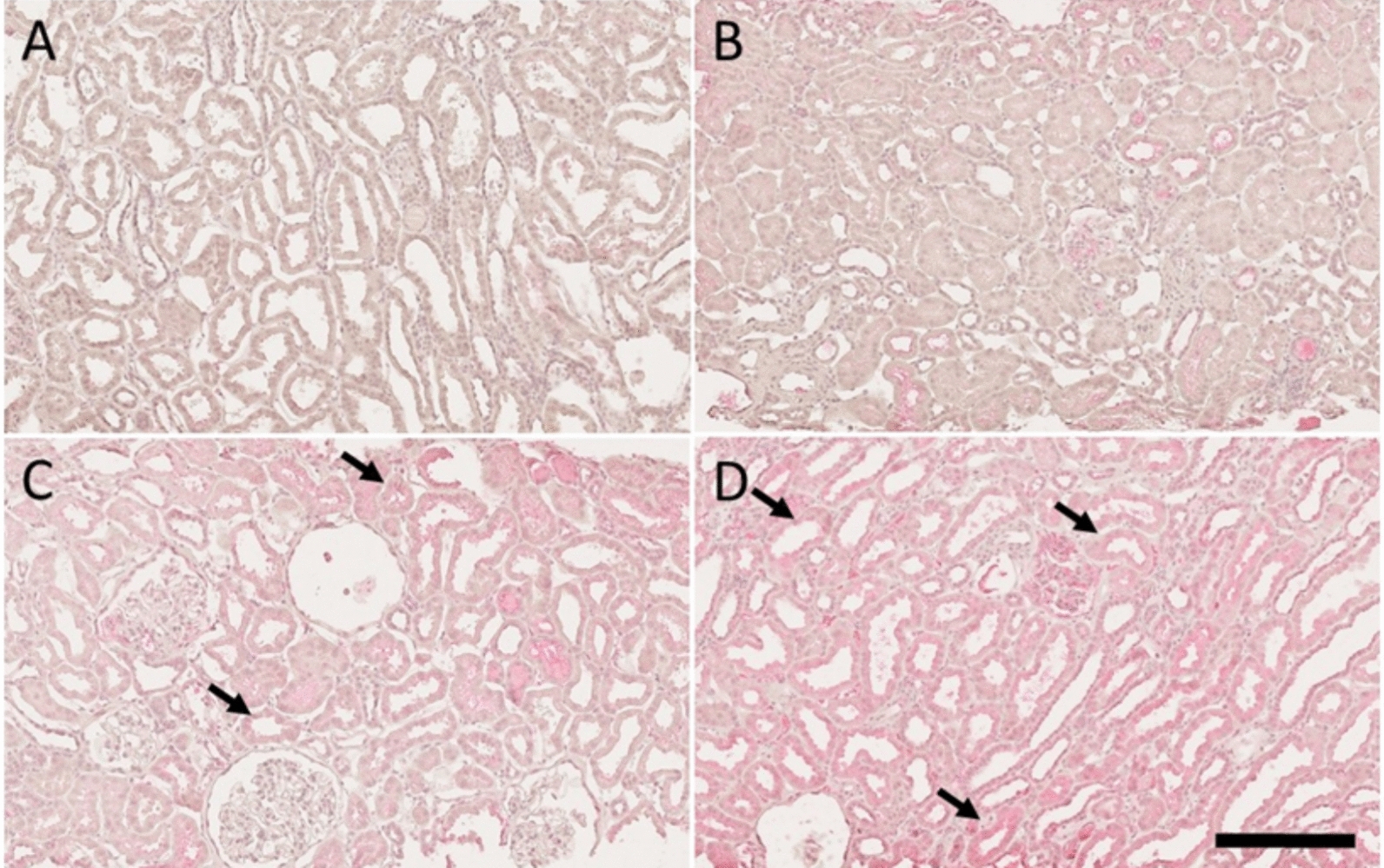
Light microscopic photographs of matrix metalloproteinase (MMP)−7, immunohistochemistry. The panels show varying degrees of MMP-7 staining, mainly in the proximal tubules. **A**: totally negative staining (0); **B**: very faint staining (trace) in some tubules; **C**: weakly positive staining (+) in some tubules, and **D**: varying, but clearly positive staining (+ +) in the proximal tubules

Since MMP-7 showed the highest regulation levels (> 14 FC, p = 5.6 × 10^–9^) in the proteomics comparison of the groups with AKI 2/3 vs no AKI after LT, we performed a validation using immunohistochemistry. Varying degrees of MMP-7 staining were found mainly in the proximal tubules (Fig. 3). A moderate correlation (r = 0.42) was found between the presence of AKI (regardless of degree) and the semi-quantitative staining assessment.

### In silico* validation*

The data were validated using 14 AKI and IRI proteomic and transcriptomic databases. First, we performed an *in-silico* validation against seven proteomics datasets. The data overlap was only partial between our data and the cohorts used. This was probably due to the broad diversity of AKI origin and AKI models studied, and the diverse sampling times. In many cases, the sampling times could not adequately mirror the timing of our biopsies. Nevertheless, comparative pathway analysis showed that cytokine signaling is a common upregulated pathway in the AKI proteomic cohorts used for validation (Supplementary Table 5). This implies that mechanisms usually found in manifest, later stages of AKI are still at their inception during the final phase of the LT procedure.

To compare our data with early-stage AKI cohorts, we extended our analysis to include 7 transcriptomic datasets (mouse IRI models and human AKI). Comparative pathway analysis confirmed the upregulation of ECM, collagen, platelet-derived growth factor (PDGF), interferons, immunity and integrin-related signaling pathways at the gene expression level. Among the downregulated pathways, five were related to mitochondrial biogenesis and energy processes. Overall, using both proteomic and transcriptomic datasets, 21 upregulated and 16 downregulated pathways identified in our study could be validated (Supplementary Table 5).

### Circulating alarmins

**Fig. 4:**
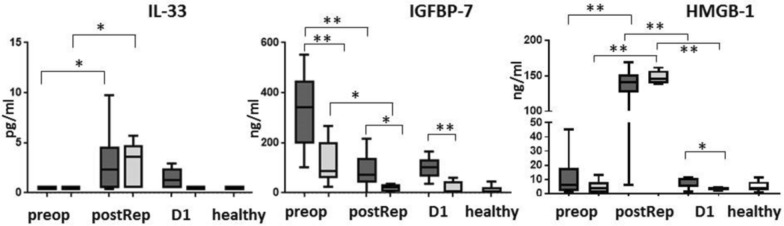
Circulating concentrations of interleukin (IL)−33, Insulin-like growth factor binding protein (IGFBP)−7, and high-mobility group box (HMGB)−1 protein preoperatively (pre), 2–4 h after liver graft reperfusion (postop), and 24 h after liver transplantation (**D1**) in the AKI group (dark grey), no AKI group (light grey), and healthy controls (n = 6, white). * p < 0.5, ** p < 0.01

Given the clear evidence of alarmin signaling in our proteomic dataset, we analysed serum levels of three known alarmins, IL-33, IGFBP-7, and HMGB-1, the latter two being detected by renal proteomics. IL-33 was undetectable preoperatively and in healthy individuals, and was therefore inputted as half LLOD (i.e., 0.45 pg/ml), whereas patients undergoing LT had higher preoperative serum IGFBP-7 compared to healthy individuals (Fig. 4). Surgery and liver graft reperfusion led to significant increases in IL-33 and HMGB-1, which returned to baseline values within the first postoperative day. IGFBP-7 decreased during LT.

## Discussion

This study found a distinct proteomic signature in the kidneys of LT recipients who went on to develop early postoperative AKI. These changes were dominated by alterations of multiple pathways involved in ECM degradation, remodeling, and inflammatory signaling, reflecting the emergence of inflammatory activity in the kidneys of LT recipients. Mitochondrial pathways were also severely downregulated in patients who developed AKI 2/3. Although AKI after LT has been the subject of considerable research, to our knowledge, this is the first study based on kidney biopsies sampled during the LT procedure to be used for histological and molecular analysis.

Postoperative development of AKI after LT has been ascribed to numerous factors related to the donor, recipient, and the transplant procedure itself, but also to mechanisms that may be driven by the newly transplanted liver graft. Previous studies indicate that a more advanced reperfusion injury is correlated with the development of postoperative AKI [38, 39]. Similarly, the current data reveal that LT recipients with AKI have worse liver function tests at every time point measured, indirectly corroborating more advanced reperfusion injury. In line with this, we recently reported that grafts of LT recipients who developed AKI have increased tissue expression of several key proteins involved in the inflammatory response and neutrophil degranulation, consistent with a more prominent reperfusion process [13].

Our study investigated the time course of three different alarmins, IL-33, HMGB-1, and IGFBP-7, as putative mediators of the inflammatory activation and subsequent remote organ injury and found notable changes in connection with LT. Alarmins are a class of endogenous molecules with immunomodulatory properties that are released or expressed by injured or necrotic cells [40]. We found significant increases in IL-33 upon liver graft reperfusion, dropping to normal values 24 h after LT. This is in line with a recent report indicating that IL-33 is immediately released as an alarmin both after murine liver recirculation and after the reperfusion of human liver grafts and appears to correlate with the severity of liver injury [41]. Remarkably, the same study found that patients with impaired renal function in the post-transplant period had higher levels of serum IL-33 upon reperfusion. Likewise, HMGB-1, which is generally regarded as the archetype of alarmins, has been found to be increased immediately after graft reperfusion, although it returned to normal values on postoperative day 1 [42] and our study revealed similar findings. Nevertheless, we could not discern any differences in the circulating levels of IL-33 and HMGB-1 between patients who developed AKI and those with an uneventful course (no AKI), likely due to the lower number of cases or imperfect sampling timing. Our interpretation of the causal roles of these two alarmins in the pathogenesis of AKI after LT is quite speculative and requires further experimental validation. In addition, we believe that the most effective intervention to counter alarmin release and signaling is mitigating ischemia–reperfusion injury in the liver graft rather than additional pharmacologic interventions targeting alarmins, which may lead to previously uncharacterized and potentially deleterious immune reactions.

On the other hand, IGFBP-7 exhibited a different, more intriguing time course. There was a low but significant increase in IGFBP-7 in the kidneys of patients who developed AKI. In previous studies, IGFBP-7 was found to be increased preoperatively in LT patients compared to healthy individuals, a finding which may be explained by the liver steatosis, fibrosis, or cirrhosis present in most of the patients [43–45]. The mechanisms for the decrease and rapid clearance of IGFBP-7 from plasma are uncertain, although sequestration in the kidney or renal loss are plausible. Increased urinary IGFBP-7 levels have been repeatedly reported following renal injury of various etiologies, to such an extent that urinary IGFBP-7 is considered a valid biomarker of kidney injury [46, 47]. Moreover, others have reported the degradation of IGFBPs by MMPs, in particular MMP-7 [48, 49], an enzyme responsible for ECM remodeling that was found to be significantly upregulated in renal biopsies obtained post-reperfusion in the present study.

ECM remodeling appeared as one of the most prominent processes ongoing in the kidneys of LT patients who developed AKI. As the ECM is affected in various kidney diseases, dysregulation of ECM dynamics is a central finding of this study. The ECM is a network of proteins and other molecules (mainly lipids and glycans) with numerous functions, including tissue architecture, cell attachment, growth and communication. The uncontrolled regulation of ECM components, such as collagens, proteoglycans, and glycoproteins (e.g. agrin, decorin, versican, biglycan, and laminins), has been described in numerous kidney diseases [50, 51]. Although the exact significance of increased MMP-7 is unclear, it is likely part of an acute response to cytokines/chemokines. Some reports suggest that MMP-7 upregulation is protective; in an animal model study, MMP-7 knockout mice experienced higher mortality and tissue injury after ischemia, whereas exogenous MMP-7 ameliorated renal harm in MMP-7 knockout mice after ischemia/reperfusion [46]. Conversely, other reports imply that MMP-7 plays an important role in the progression of ischemic renal injury by activating MMP-2 and −9 and by disturbing the integrity of the tubular epithelium through the degradation of tight junctional proteins [52]. The mechanical, supportive role of the ECM is influenced by various circulating mediators, with consequences on organ functionality. For instance, integrin signaling regulates, in part, ECM dynamics and is intimately related to cell shape and function. Expectedly, the integrin cell surface interactions pathway was among those upregulated in the group of patients with AKI 2/3 after LT. Podocytes, for example, are affected by integrin fiber thickness variations [53], and cytokine signaling can trigger the secretion of large amounts of ECM and pave the way for interstitial fibrosis and later CKD [54]. Conversely, PDGF signaling is associated with inflammasome activation and lipid variations underlying inflammatory stimuli in glomerular mesangial cells [55]. Our proteomics data show that 11 out of 18 detected collagen isoforms (which are important ECM components) were upregulated in LT patients with AKI (data not shown). Furthermore, two proteins related to fibrosis, such as fibronectin 1 and transforming growth factor beta receptor I showed an upregulation trend. TGFβ signaling, particularly in injured proximal tubular cells, is suggested to be a mediator of the transition from AKI to CKD [56]. Increased collagen production is also typical of CKD, and a recent assessment of collagen turnover found it to be well aligned with CKD severity [57]. Hence, altogether, these findings may represent the initial triggering events that eventually lead to CKD, which is frequently seen in LT patients with initial AKI [11]. Increased ECM-receptor interactions have been indicated as a predictor of AKI in kidney transplant patients [58], whereas tissue inhibitor of metalloproteinases (TIMP)−2 is an acknowledged marker of kidney injury, likely reflecting ongoing ECM remodeling [59] and cell cycle arrest in response to cellular stress. In this context, we speculate that some of the ECM alterations could also be attributed to IL-33, which, in addition to its known pro-inflammatory profile, is likely involved in ECM remodeling and MMP activation [59]. Moreover, IL-33 has an unclear but harmful effect on renal tubular epithelial cells, which has been described in kidney transplantation [60]. A strong positive correlation was found between IL-33 levels and MMP-1 and MMP-2 and their inhibitors TIMP-1 and −2 in mitral valves with myxomatous degeneration, suggesting the direct involvement of IL-33 in the remodeling of valvular ECM [61]. The disruption of the basement membrane (tubular and glomerular) and the ECM via cytokine-induced alterations in MMPs and TIMPs is well established [62, 63]. We believe that the intraoperative IL-33 surge and its tendency towards longer persistence in patients with AKI could also have directly contributed to the ECM alterations noted herein, as well as in the occurrence of early AKI.

Another key finding of our study was the dysregulation of mitochondrial pathways in AKI after LT. Mitochondria are the principal source of cellular energy, and paradoxically, the supply of many substances that can cause cell damage or death. Mitochondrial injury appears to precede the clinical evidence of AKI. Different harmful mechanisms related to AKI, both ischemic and toxic, cause impairment of mitochondrial function. Cellular harm is further enhanced by the mitochondrial response to cellular stress. This results at worst in reactive oxygen species (ROS) production or caspase activation and consequently apoptosis [62]. Impairment of mitochondrial functions and ATP production was described after 24 h of IRI in a rat model in one of the studies used for validation, in agreement with our data [37]. In particular, subunits and accessory proteins of the NADH ubiquinone oxidoreductase, electron transport chain complex I, are significantly depleted. Of note, disruption of renal mitochondrial dynamics was previously reported in a rat model of orthotopic LT [63]. Remarkably, the histological findings were discrete and identical with those described herein (loss of brush border, vacuolization of tubular cells in the cortex). In that study, mitochondrial fission capacity was found to be heavily decreased, mitochondrial DNA copy number dropped by 64%, whereas mitophagy was greatly increased. Of note, these results suggesting ongoing severe mitochondrial impairment following LT were obtained from rat kidneys that were sampled 18 h after LT. This could imply that energetic failure could extend into, and drive the later stages of AKI.

We believe that the proteomic changes reported herein, which contribute to the initiation and increase the impact of early post-LT AKI, primarily occurred in the interval between the first biopsy, performed at the start of LT, and the second biopsy obtained soon after liver graft reperfusion. However, differences were already present between the protein signatures of biopsies obtained at baseline and those obtained at the start of LT. These differences are related to inflammatory and mitochondrial processes that are activated in the post-reperfusion biopsies, which most probably prime the kidneys for further injurious signaling at reperfusion. Moreover, the proteomic evolution in the AKI 2/3 group during the LT procedure further enhances the impression of accentuated inflammation and altered ECM dynamics. Although it remains unclear which event triggers these intraoperative renal alterations, we believe that graft reperfusion has the greatest potential to initiate remote organ injury through the release of alarmins, cytokines, and other proinflammatory mediators [64–66].

This study has several limitations that need to be acknowledged, with the most obvious being the relatively low sample size of the groups. Logistic and cost-related reasons prevented us from analyzing samples from more patients. However, studies using groups of similar sizes are frequent and widely accepted in the field of omics technologies, given the wealth of data generated simultaneously, which can be subsequently corroborated and rendered meaningful by using artificial intelligence and large external databases [67–69]. Another potential limitation is selection bias, as the analysis included only moderate and severe AKI cases, whereas samples from patients with milder AKI were not analyzed. This reasoning was based on the assumption that milder AKI may have more elusive functional causes rather than the well-defined molecular basis in more advanced AKI cases. Other potential limitations are the well-known heterogeneity of human samples and variations in the impact of their background disease on the molecular landscape in the kidney [69, 70]. Circulating proteins in the blood that may have originated in other organs could have been trapped in the biopsied kidney tissue and “contaminated” the kidney biopsy [71]. All these risks are likely equally distributed among all the samples and reflect the “real-world” setting.

In conclusion, the kidneys of LT patients who go on to develop early AKI exhibit complex proteome alterations very early after liver graft reperfusion. These changes involve ECM remodeling and mitochondrial impairment and may be the first stages of the remote organ injury observed after hepatic IRI, possibly reflecting ongoing alarmin and cytokine signaling.

## Supplementary Information

Suplementary material 1. Table 1. All regulated proteins in the AKI 2/3 vs no AKI groups, comparison after transplantation. AKI, acute kidney injury

Suplementary material 2. Table 2. Top 100 most up- and downregulated proteins in the AKI vs no AKI groups, comparison before transplantation. AKI, acute kidney injury; FC, fold change

Suplementary material 3. Table 3. Most differentially regulated pathways in the AKI vs no AKI groups, comparison before transplantation. AKI, acute kidney injury; FDR, false discovery rate

Suplementary material 4. Table 4. All regulated proteins in the AKI group, before vs after transplantation. AKI, acute kidney injury

Suplementary material 5. Table 5. Material used for proteomics and transcriptomics validation. For each dataset used in the in silico validation, we report the article reference, analysis type, the source of material and species, and the dataset used for comparison

## Data Availability

Data on which the study is based can be made available upon reasonable request, unless it would breach patient confidentiality.

## References

[1] Kwong AJ, Ebel NH, Kim WR, Lake JR, Smith JM, Schladt DP, Skeans MA, Foutz J, Gauntt K, Cafarella M, Snyder JJ, Israni AK, Kasiske BL. OPTN/SRTR 2020 annual data report: liver. Am J Trans. 2022;22(Suppl 2):204–309. 10.1111/ajt.16978 35266621

[2] Nadim MK, Genyk YS, Tokin C, Fieber J, Ananthapanyasut W, Ye W, Selby R. Impact of the etiology of acute kidney injury on outcomes following liver transplantation: acute tubular necrosis versus hepatorenal syndrome. Liver Transpl. 2012;18:539–48. 22250075 10.1002/lt.23384

[3] Ojo AO, Held PJ, Port FK, Wolfe RA, Leichtman AB, Young EW, Arndorfer J, Christensen L, Merion RM. Chronic renal failure after transplantation of a nonrenal organ. N Engl J Med. 2003;349(10):931–40. 12954741 10.1056/NEJMoa021744

[4] Asrani SK, Shankar N, da Graca B, Nadim MK, Cardenas A. Role of novel kidney biomarkers in patients with cirrhosis and after liver transplantation. Liver Transpl. 2022;28(3):466–82. 34714972 10.1002/lt.26344

[5] Niemann CU, Walia A, Waldman J, Davio M, Roberts JP, Hirose R, Feiner J. Acute kidney injury during liver transplantation as determined by neutrophil gelatinase-associated lipocalin. Liver Transpl. 2009;15(12):1852–60. 19938135 10.1002/lt.21938

[6] Cheng CW, Chen YC, Chang CH, Yu HP, Lin CC, Yang MW, Lee WC, Chang CJ. The ratio of plasma neutrophil gelatinase-associated lipocalin predicts acute kidney injury in patients undergoing liver transplantation. Transplant Proc. 2012;44(3):776–9. 22483493 10.1016/j.transproceed.2012.01.068

[7] Jeong TD, Kim S, Lee W, Song GW, Kim YK, Chun S, Lee SG, Min WK. Neutrophil gelatinase-associated lipocalin as an early biomarker of acute kidney injury in liver transplantation. Clin Trans. 2012;26(5):775–81. 10.1111/j.1399-0012.2012.01610.x 22404749

[8] Yeung ACY, Morozov A, Robertson FP, Fuller BJ, Davidson BR. Neutrophil gelatinase-associated lipocalin (NGAL) in predicting acute kidney injury following orthotopic liver transplantation: a systematic review. Int J Surg. 2018;59:48–54. 30273683 10.1016/j.ijsu.2018.09.003

[9] Barri YM, Sanchez EQ, Jennings LW, Melton LB, Hays S, Levy MF, Klintmalm GB. Acute kidney injury following liver transplantation: definition and outcome. Liver Transpl. 2009;15(5):475–83. 19399734 10.1002/lt.21682

[10] Hilmi IA, Damian D, Al-Khafaji A, Planinsic R, Boucek C, Sakai T, Chang CC, Kellum JA. Acute kidney injury following orthotopic liver transplantation: incidence, risk factors, and effects on patient and graft outcomes. Br J Anaesth. 2015;114(6):919–26. 25673576 10.1093/bja/aeu556

[11] Norén Å, Åberg F, Mölne J, Bennet W, Friman S, Herlenius G. Perioperative kidney injury in liver transplantation: a prospective study with renal histology and measured glomerular filtration rates. Scand J Gastroenterol. 2022;57(5):595–602. 35060823 10.1080/00365521.2022.2028004

[12] Skytte Larsson J, Bragadottir G, Redfors B, Ricksten SE. Renal function and oxygenation are impaired early after liver transplantation despite hyperdynamic systemic circulation. Crit Care. 2017;21(1):87. 28395663 10.1186/s13054-017-1675-4 PMC5387193

[13] Norén Å, Oltean M, Friman S, Molinaro A, Mölne J, Sihlbom C, Herlenius G, Thorsell A. Liver graft proteomics reveals potential incipient mechanisms behind early renal dysfunction after liver transplantation. int J Mol Sci. 2022;23(19):11929. 36233231 10.3390/ijms231911929 PMC9569532

[14] de Haan JE, Hoorn EJ, de Geus HRH. Acute kidney injury after liver transplantation: recent insights and future perspectives. Best Pract Res Clin Gastroenterol. 2017;31(2):161–9. 28624104 10.1016/j.bpg.2017.03.004

[15] Hukriede NA, Soranno DE, Sander V, Perreau T, Starr MC, Yuen PST, Siskind LJ, Hutchens MP, Davidson AJ, Burmeister DM, Faubel S, de Caestecker MP. Experimental models of acute kidney injury for translational research. Nat Rev Nephrol. 2022;18:277–93. 35173348 10.1038/s41581-022-00539-2 PMC12439461

[16] Yin W, Zhang PL, Macknis JK, Lin F, Bonventre JV. Kidney injury molecule-1 identifies antemortem injury in postmortem adult and fetal kidney. Am J Physiol Renal Physiol. 2018;315(6):F1637–43. 30110569 10.1152/ajprenal.00060.2018 PMC6337001

[17] Fiorentino M, Kellum JA. Improving translation from preclinical studies to clinical trials in acute kidney injury. Nephron. 2018;140(2):81–5. 29791911 10.1159/000489576

[18] Khwaja A. KDIGO clinical practice guidelines for acute kidney injury. Nephron Clin Pract. 2012;120(4):c179–84. 22890468 10.1159/000339789

[19] Roufosse C, Simmonds N, Clahsen-van Groningen M, Haas M, Henriksen KJ, Horsfield C, Loupy A, Mengel M, Perkowska-Ptasińska A, Rabant M, Racusen LC, Solez K, Becker JU. A 2018 reference guide to the banff classification of renal allograft pathology. Transplantation. 2018;102(11):1795–814. 30028786 10.1097/TP.0000000000002366 PMC7597974

[20] Goujon JM, Hauet T, Menet E, Levillain P, Babin P, Carretier M. Histological evaluation of proximal tubule cell injury in isolated perfused pig kidneys exposed to cold ischemia. J Surg Res. 1999;82(2):228–33. 10090834 10.1006/jsre.1998.5526

[21] Crescitelli R, Lässer C, Jang SC, Cvjetkovic A, Malmhäll C, Karimi N, Höög JL, Johansson I, Fuchs J, Thorsell A, Gho YS, Olofsson Bagge R, Lötvall J. Subpopulations of extracellular vesicles from human metastatic melanoma tissue identified by quantitative proteomics after optimized isolation. J Extracell Vesicles. 2020;9(1):1722433. 32128073 10.1080/20013078.2020.1722433 PMC7034452

[22] Dubois E, Galindo AN, Dayon L, Cominetti O. Assessing normalization methods in mass spectrometry-based proteome profiling of clinical samples. Biosystems. 2022;215–216: 104661. 35247480 10.1016/j.biosystems.2022.104661

[23] Ritchie ME, Phipson B, Wu D, Hu Y, Law CW, Shi W, Smyth GK. limma powers differential expression analyses for RNA-sequencing and microarray studies. Nucl Acids Res. 2015;43(7): e47. 25605792 10.1093/nar/gkv007 PMC4402510

[24] Aregger F, Uehlinger DE, Witowski J, Brunisholz RA, Hunziker P, Frey FJ, Jörres A. Identification of IGFBP-7 by urinary proteomics as a novel prognostic marker in early acute kidney injury. Kidney Int. 2014;85(4):909–19. 24067438 10.1038/ki.2013.363

[25] Correa-Costa M, Azevedo H, Amano MT, Gonçalves GM, Hyane MI, Cenedeze MA, Renesto PG, Pacheco-Silva A, Moreira-Filho CA, Câmara NO. Transcriptome analysis of renal ischemia/reperfusion injury and its modulation by ischemic pre-conditioning or hemin treatment. PLoS ONE. 2012;7(11): e49569. 23166714 10.1371/journal.pone.0049569 PMC3498198

[26] Daniels JR, Ma JZ, Cao Z, Beger RD, Sun J, Schnackenberg L, Pence L, Choudhury D, Palevsky PM, Portilla D, Yu LR. Discovery of novel proteomic biomarkers for the prediction of kidney recovery from dialysis-dependent AKI patients. Kidney360. 2021;2(11):1716–27. 34913041 10.34067/KID.0002642021 PMC8670726

[27] Dixon EE, Wu H, Muto Y, Wilson PC, Humphreys BD. Spatially resolved transcriptomic analysis of acute kidney injury in a female murine model. J Am Soc Nephrol. 2022;33(2):279–89. 34853151 10.1681/ASN.2021081150 PMC8819997

[28] Gerhardt LMS, Liu J, Koppitch K, Cippà PE, McMahon AP. Single-nuclear transcriptomics reveals diversity of proximal tubule cell states in a dynamic response to acute kidney injury. Proc Natl Acad Sci USA. 2021;118(27): e2026684118. 34183416 10.1073/pnas.2026684118 PMC8271768

[29] Hinze C, Kocks C, Leiz J, Karaiskos N, Boltengagen A, Cao S, Skopnik CM, Klocke J, Hardenberg JH, Stockmann H, Gotthardt I, Obermayer B, Haghverdi L, Wyler E, Landthaler M, Bachmann S, Hocke AC, Corman V, Busch J, Schneider W, Himmerkus N, Bleich M, Eckardt KU, Enghard P, Rajewsky N, Schmidt-Ott KM. Single-cell transcriptomics reveals common epithelial response patterns in human acute kidney injury. Genome Med. 2022;14(1):103. 36085050 10.1186/s13073-022-01108-9 PMC9462075

[30] Jin J, Gong J, Zhao L, Li Y, Wang Y, He Q. iTRAQ-based comparative proteomics analysis reveals specific urinary biomarkers for various kidney diseases. Biomark Med. 2020;14(10):839–54. 32856461 10.2217/bmm-2019-0556

[31] Kirita Y, Wu H, Uchimura K, Wilson PC, Humphreys BD. Cell profiling of mouse acute kidney injury reveals conserved cellular responses to injury. Proc Natl Acad Sci USA. 2020;117(27):15874–83. 32571916 10.1073/pnas.2005477117 PMC7355049

[32] Lin YH, Platt MP, Fu H, Gui Y, Wang Y, Gonzalez-Juarbe N, Zhou D, Yu Y. Global proteome and phosphoproteome characterization of sepsis-induced kidney injury. Mol Cell Proteom. 2020;19(12):2030–47. 10.1074/mcp.RA120.002235 PMC7710145 32963032

[33] Malagrino PA, Venturini G, Yogi PS, Dariolli R, Padilha K, Kiers B, Gois TC, Cardozo KH, Carvalho VM, Salgueiro JS, Girardi AC, Titan SM, Krieger JE, Pereira AC. Proteome analysis of acute kidney injury—discovery of new predominantly renal candidates for biomarker of kidney disease. J Proteomics. 2017;151:66–73. 27457269 10.1016/j.jprot.2016.07.019

[34] Melo Ferreira R, Sabo AR, Winfree S, Collins KS, Janosevic D, Gulbronson CJ, Cheng YH, Casbon L, Barwinska D, Ferkowicz MJ, Xuei X, Zhang C, Dunn KW, Kelly KJ, Sutton TA, Hato T, Dagher PC, El-Achkar TM, Eadon MT. Integration of spatial and single-cell transcriptomics localizes epithelial cell-immune cross-talk in kidney injury. JCI Insight. 2021;6(12): e147703. 34003797 10.1172/jci.insight.147703 PMC8262485

[35] Paranjpe I, Jayaraman P, Su CY, Zhou S, Chen S, Thompson R, Del Valle DM, Kenigsberg E, Zhao S, Jaladanki S, Chaudhary K, Ascolillo S, Vaid A, Kumar A, Kozlova E, Paranjpe M, O’Hagan R, Kamat S, Gulamali FF, Kauffman J, Xie H, Harris J, Patel M, Argueta K, Batchelor C, Nie K, Dellepiane S, Scott L, Levin MA, He JC, Suarez-Farinas M, Coca SG, Chan L, Azeloglu EU, Schadt E, Beckmann N, Gnjatic S, Merad M, Kim-Schulze S, Richards B, Glicksberg BS, Charney AW, Nadkarni GN. Proteomic characterization of acute kidney injury in patients hospitalized with sars-cov2 infection. Medrxiv. 2022;29:2021. 10.1101/2021.12.09.21267548.

[36] Park M, Kwon CH, Ha HK, Han M, Song SH. RNA-Seq identifies condition-specific biological signatures of ischemia-reperfusion injury in the human kidney. BMC Nephrol. 2020;21(Suppl 1):398. 32977749 10.1186/s12882-020-02025-y PMC7517631

[37] Huang H, van Dullemen LFA, Akhtar MZ, Faro ML, Yu Z, Valli A, Dona A, Thézénas ML, Charles PD, Fischer R, Kaisar M, Leuvenink HGD, Ploeg RJ, Kessler BM. Proteo-metabolomics reveals compensation between ischemic and non-injured contralateral kidneys after reperfusion. Sci Rep. 2018;8(1):8539. 29867102 10.1038/s41598-018-26804-8 PMC5986744

[38] Leithead JA, Rajoriya N, Gunson BK, Muiesan P, Ferguson JW. The evolving use of higher risk grafts is associated with an increased incidence of acute kidney injury after liver transplantation. J Hepatol. 2014;60(6):1180–6. 24631601 10.1016/j.jhep.2014.02.019

[39] Jochmans I, Meurisse N, Neyrinck A, Verhaegen M, Monbaliu D, Pirenne J. Hepatic ischemia/reperfusion injury associates with acute kidney injury in liver transplantation: prospective cohort study. Liver Transpl. 2017;23(5):634–44. 28124458 10.1002/lt.24728

[40] Sabapathy V, Venkatadri R, Dogan M, Sharma R. The Yin and Yang of Alarmins in regulation of acute kidney injury. Front Med. 2020;21(7):441. 10.3389/fmed.2020.00441 PMC7472534 32974364

[41] Barbier L, Robin A, Sindayigaya R, Ducousso H, Dujardin F, Thierry A, Hauet T, Girard JP, Pellerin L, Gombert JM, Herbelin A, Salamé E. Endogenous interleukin-33 acts as an alarmin in liver Ischemia-reperfusion and is associated with injury after human liver transplantation. Front Immunol. 2021;12: 744927. 34621275 10.3389/fimmu.2021.744927 PMC8491545

[42] Sosa RA, Terry AQ, Kaldas FM, Jin YP, Rossetti M, Ito T, Li F, Ahn RS, Naini BV, Groysberg VM, Zheng Y, Aziz A, Nevarez-Mejia J, Zarrinpar A, Busuttil RW, Gjertson DW, Kupiec-Weglinski JW, Reed EF. Disulfide high-mobility group box 1 drives ischemia-reperfusion injury in human liver transplantation. Hepatology. 2021;73(3):1158–75. 32426849 10.1002/hep.31324 PMC8722704

[43] Fourman LT, Stanley TL, Ockene MW, McClure CM, Toribio M, Corey KE, Chung RT, Torriani M, Kleiner DE, Hadigan CM, Grinspoon SK. Proteomic analysis of hepatic fibrosis in human immunodeficiency virus-associated nonalcoholic fatty liver disease demonstrates up-regulation of immune response and tissue repair pathways. J Infect Dis. 2023;227(4):565–76. 36461941 10.1093/infdis/jiac475 PMC10152500

[44] Martínez-Castillo M, Rosique-Oramas D, Medina-Avila Z, Pérez-Hernández JL, Higuera-De la Tijera F, Santana-Vargas D, Montalvo-Jave EE, Sanchez-Avila F, Torre A, Kershenobich D, Gutierrez-Reyes G. Differential production of insulin-like growth factor-binding proteins in liver fibrosis progression. Mol Cell Biochem. 2020;469(1–2):65–75. 32301061 10.1007/s11010-020-03728-4

[45] Stanley TL, Fourman LT, Zheng I, McClure CM, Feldpausch MN, Torriani M, Corey KE, Chung RT, Lee H, Kleiner DE, Hadigan CM, Grinspoon SK. Relationship of IGF-1 and IGF-binding proteins to disease severity and glycemia in nonalcoholic fatty liver disease. J Clin Endocrinol Metab. 2021;106(2):e520–33. 33125080 10.1210/clinem/dgaa792 PMC7823253

[46] Westhoff JH, Tönshoff B, Waldherr S, Pöschl J, Teufel U, Westhoff TH, Fichtner A. Urinary tissue inhibitor of metalloproteinase-2 (TIMP-2) • insulin-like growth factor-binding protein 7 (IGFBP7) predicts adverse outcome in pediatric acute kidney injury. PLoS ONE. 2015;10(11): e0143628. 26606754 10.1371/journal.pone.0143628 PMC4659607

[47] Molinari L, Del Rio-Pertuz G, Smith A, Landsittel DP, Singbartl K, Palevsky PM, Chawla LS, Huang DT, Yealy DM, Angus DC, Kellum JA, ProCESS ProGReSS-AKI Investigators. Utility of biomarkers for sepsis-associated acute kidney injury staging. JAMA Netw Open. 2022;5(5):2212709. 10.1001/jamanetworkopen.2022.12709 PMC9118077 35583867

[48] Miyamoto S, Yano K, Sugimoto S, Ishii G, Hasebe T, Endoh Y, Kodama K, Goya M, Chiba T, Ochiai A. Matrix metalloproteinase-7 facilitates insulin-like growth factor bioavailability through its proteinase activity on insulin-like growth factor binding protein 3. Cancer Res. 2004;64(2):665–71. 14744783 10.1158/0008-5472.can-03-1916

[49] Jin L, Shen F, Weinfeld M, Sergi C. Insulin growth factor binding protein 7 (IGFBP7)-related cancer and IGFBP3 and IGFBP7 crosstalk. Front Oncol. 2020;10:727. 32500027 10.3389/fonc.2020.00727 PMC7242731

[50] Tan RJ, Liu Y. Matrix metalloproteinases in kidney homeostasis and diseases: an update. Am J Physiol Renal Physiol. 2024;327(6):F967–84. 39361724 10.1152/ajprenal.00179.2024 PMC11687849

[51] Bülow RD, Boor P. Extracellular matrix in kidney fibrosis: more than just a scaffold. J Histochem Cytochem. 2019;67(9):643–61. 31116062 10.1369/0022155419849388 PMC6713975

[52] Fu H, Zhou D, Zhu H, Liao J, Lin L, Hong X, Hou FF, Liu Y. Matrix metalloproteinase-7 protects against acute kidney injury by priming renal tubules for survival and regeneration. Kidney Int. 2019;95(5):1167–80. 30878215 10.1016/j.kint.2018.11.043 PMC6478554

[53] Hu Q, Lan J, Liang W, Chen Y, Chen B, Liu Z, Xiong Y, Zhong Z, Wang Y, Ye Q. MMP7 damages the integrity of the renal tubule epithelium by activating MMP2/9 during ischemia-reperfusion injury. J Mol Histol. 2020;51(6):685–700. 33070277 10.1007/s10735-020-09914-4

[54] Boi R, Bergwall L, Ebefors K, Bergö MO, Nyström J, Buvall L. Podocyte geranylgeranyl transferase type-I Is essential for maintenance of the glomerular filtration barrier. J Am Soc Nephrol. 2023;34(4):641–55. 36735952 10.1681/ASN.0000000000000062 PMC10103324

[55] Liang Y, Qu L, Liu Z, Liang L, Wang Y, Quan S, Wang Y, Tang L. The IRE1/JNK signaling pathway regulates inflammation cytokines and production of glomerular extracellular matrix in the acute kidney injury to chronic kidney disease transition. Mol Biol Rep. 2022;49(8):7709–18. 35696049 10.1007/s11033-022-07588-7

[56] Boi R, Ebefors K, Henricsson M, Borén J, Nyström J. Modified lipid metabolism and cytosolic phospholipase A2 activation in mesangial cells under pro-inflammatory conditions. Sci Rep. 2022;12(1):7322. 35513427 10.1038/s41598-022-10907-4 PMC9072365

[57] Gewin LS. Transforming growth factor-β in the acute kidney injury to chronic kidney disease transition. Nephron. 2019;143(3):154–7. 31039574 10.1159/000500093 PMC6821554

[58] Rasmussen DGK, Boesby L, Nielsen SH, Tepel M, Birot S, Karsdal MA, Kamper AL, Genovese F. Collagen turnover profiles in chronic kidney disease. Sci Rep. 2019;9(1):16062. 31690732 10.1038/s41598-019-51905-3 PMC6831687

[59] Zhai X, Lou H, Hu J. Five-gene signature predicts acute kidney injury in early kidney transplant patients. Aging. 2022;14(6):2628–44. 35320116 10.18632/aging.203962 PMC9004575

[60] Ariyoshi W, Okinaga T, Chaweewannakorn W, Akifusa S, Nisihara T. Mechanisms involved in enhancement of matrix metalloproteinase-9 expression in macrophages by interleukin-33. J Cell Physiol. 2017;232(12):3481–95. 28105703 10.1002/jcp.25809

[61] Ferhat M, Robin A, Giraud S, Sena S, Goujon JM, Touchard G, Hauet T, Girard JP, Gombert JM, Herbelin A, Thierry A. Endogenous IL-33 Contributes to Kidney Ischemia-Reperfusion Injury as an Alarmin. J Am Soc Nephrol. 2018;29(4):1272–88. 29436517 10.1681/ASN.2017060650 PMC5875946

[62] Garcia-Pena A, Ibarrola J, Navarro A, Sadaba A, Tiraplegui C, Garaikoetxea M, Arrieta V, Matilla L, Fernández-Celis A, Sadaba R, Alvarez V, Gainza A, Jover E, López-Andrés N. Activation of the interleukin-33/ST2 pathway exerts deleterious effects in myxomatous mitral valve disease. Int J Mol Sci. 2021;22(5):2310. 33669101 10.3390/ijms22052310 PMC7956196

[63] Liu Q, Krishnasamy Y, Rehman H, Lemasters JJ, Schnellmann RG, Zhong Z. Disrupted renal mitochondrial homeostasis after liver transplantation in rats. PLoS ONE. 2015;10(10): e0140906. 26480480 10.1371/journal.pone.0140906 PMC4610703

[64] Nee LE, McMorrow T, Campbell E, Slattery C, Ryan MP. TNF-alpha and IL-1beta-mediated regulation of MMP-9 and TIMP-1 in renal proximal tubular cells. Kidney Int. 2004;66(4):1376–86. 15458430 10.1111/j.1523-1755.2004.00900.x

[65] Nee L, Tuite N, Ryan MP, McMorrow T. TNF-alpha and IL-1 beta-mediated regulation of MMP-9 and TIMP-1 in human glomerular mesangial cells. Nephron Exp Nephrol. 2007;107(2):e73-86. 17890880 10.1159/000108645

[66] Sosa RA, Zarrinpar A, Rossetti M, Lassman CR, Naini BV, Datta N, Rao P, Harre N, Zheng Y, Spreafico R, Hoffmann A, Busuttil RW, Gjertson DW, Zhai Y, Kupiec-Weglinski JW, Reed EF. Early cytokine signatures of ischemia/reperfusion injury in human orthotopic liver transplantation. JCI Insight. 2016;1(20): e89679. 27942590 10.1172/jci.insight.89679 PMC5135282

[67] Kip AM, Valverde JM, Altelaar M, Heeren RMA, Hundscheid IHR, Dejong CHC, Olde Damink SWM, Balluff B, Lenaerts K. Combined quantitative (phospho)proteomics and mass spectrometry imaging reveal temporal and spatial protein changes in human intestinal ischemia-reperfusion. J Proteome Res. 2022;21(1):49–66. 34874173 10.1021/acs.jproteome.1c00447 PMC8750167

[68] Alicehajic A, Duivenvoorden AAM, Lenaerts K. Unveiling the molecular complexity of intestinal ischemia-reperfusion injury through omics technologies. Proteomics. 2024;24(12–13): e2300160. 38477684 10.1002/pmic.202300160

[69] Acharya P, Saha R, Quadri JA, Sarwar S, Khan MA, Sati HC, Gauniyal N, Shariff A, Swaroop S, PathakShalimar P. Quantitative plasma proteomics identifies metallothioneins as a marker of acute-on-chronic liver failure associated acute kidney injury. Front Immunol. 2023;13:1041230. 36776389 10.3389/fimmu.2022.1041230 PMC9909472

[70] Allegretti AS, Solà E, Ginès P. Clinical application of kidney biomarkers in cirrhosis. Am J Kidney Dis. 2020;76(5):710–9. 32622560 10.1053/j.ajkd.2020.03.016

[71] Kollikowski AM, Pham M, März AG, Feick J, Vogt ML, Xiong Y, Strinitz M, Vollmuth C, Essig F, Neugebauer H, Haeusler KG, Hametner C, Zimmermann L, Stoll G, Schuhmann MK. MMP-9 release into collateral blood vessels before endovascular thrombectomy to assess the risk of major intracerebral haemorrhages and poor outcome for acute ischaemic stroke: a proof-of-concept study. EBioMedicine. 2024;103: 105095. 38579365 10.1016/j.ebiom.2024.105095 PMC11002809

