# Supplementary material for: Proteomic analysis of human kidney biopsies unveils emerging acute kidney injury very early after liver graft reperfusion
Source: J Transl Med. 2025 Jun 16;23:658. doi: 10.1186/s12967-025-06695-w (PMC12172208; doi:10.1186/s12967-025-06695-w)
Supplement: Supplementary file 1 — Suplementary material 1. Table 1. All regulated proteins in the AKI 2/3 vs no AKI groups, comparison after transplantation. AKI, acute kidney injury [file 12967_2025_6695_MOESM1_ESM.docx]

**Supplementary table 1.** All regulated proteins in the AKI 2/3 vs no AKI groups, comparison after transplantation

| **PROTEIN SYMBOL** | **p-value** | **Fold change** |
| --- | --- | --- |
| **MMP7** | 5.59E-09 | 14.16957 |
| **TPSAB1, TPSB2** | 1.91E-05 | 5.04853 |
| **FAM21C, WASHC2C** | 1.73E-02 | 3.44928 |
| **PROM1** | 6.68E-04 | 3.31629 |
| **MUC13** | 3.72E-03 | 2.8894 |
| **MRC1** | 1.75E-06 | 2.72971 |
| **IGJ, JCHAIN** | 8.12E-03 | 2.62565 |
| **SFT2D3** | 5.64E-03 | 2.51973 |
| **PTGIS** | 6.84E-06 | 2.3913 |
| **ADH4** | 4.55E-03 | 2.38018 |
| **MYCBP** | 4.29E-04 | 2.36493 |
| **SLC39A9** | 6.22E-03 | 2.35062 |
| **COL6A6** | 4.35E-02 | 2.3288 |
| **CRYAB** | 2.72E-04 | 2.31692 |
| **ITGB6** | 5.66E-07 | 2.25005 |
| **HIST1H1D** | 4.07E-03 | 2.225 |
| **HIST1H1B** | 7.36E-04 | 2.1916 |
| **NQO1** | 6.46E-03 | 2.10098 |
| **NT5C1A** | 3.60E-04 | 2.05229 |
| **SPP1** | 4.05E-03 | 2.04366 |
| **AKR1B10** | 1.58E-02 | 2.00452 |
| **UMOD** | 5.78E-04 | 1.99629 |
| **COL14A1** | 5.92E-03 | 1.97102 |
| **TMPRSS4** | 6.07E-04 | 1.93122 |
| **RAB3A** | 1.09E-06 | 1.92706 |
| **IGLC7** | 1.08E-02 | 1.91821 |
| **CPA3** | 7.31E-03 | 1.90993 |
| **CMA1** | 8.65E-03 | 1.90074 |
| **TOR4A** | 3.07E-04 | 1.89952 |
| **ABCC3** | 5.33E-05 | 1.89392 |
| **CES1** | 5.81E-03 | 1.89059 |
| **GDF15** | 8.01E-06 | 1.88191 |
| **ADH1A** | 4.74E-02 | 1.8774 |
| **RASAL1** | 1.79E-05 | 1.87209 |
| **TNXB** | 9.30E-06 | 1.87191 |
| **SERPINE2** | 1.19E-04 | 1.83155 |
| **C9** | 3.19E-03 | 1.80677 |
| **SPON1** | 7.99E-05 | 1.78916 |
| **SFRP1** | 2.88E-02 | 1.77735 |
| **SLC5A8** | 3.95E-03 | 1.76191 |
| **LTBP2** | 1.34E-03 | 1.74804 |
| **FBLN5** | 2.46E-03 | 1.74332 |
| **MRC2** | 2.50E-03 | 1.73739 |
| **CKMT1B, CKMT1A** | 4.32E-05 | 1.72416 |
| **PDLIM3** | 4.88E-02 | 1.72316 |
| **MYBBP1A** | 3.70E-06 | 1.72248 |
| **VKORC1** | 1.18E-02 | 1.72152 |
| **LYVE1** | 2.54E-04 | 1.71871 |
| **UCHL1** | 9.96E-04 | 1.71577 |
| **FERMT1** | 7.03E-03 | 1.71388 |
| **ARHGEF28** | 4.19E-03 | 1.70829 |
| **LRRFIP1** | 1.76E-04 | 1.69516 |
| **PIGR** | 1.53E-02 | 1.69029 |
| **CXCL14** | 2.87E-02 | 1.68675 |
| **TGM2** | 7.90E-04 | 1.68517 |
| **EFEMP2** | 7.38E-04 | 1.67534 |
| **LSP1** | 1.86E-04 | 1.65898 |
| **OAF** | 1.39E-03 | 1.65563 |
| **FRMPD1** | 4.44E-04 | 1.65406 |
| **FAM83H** | 3.27E-03 | 1.65305 |
| **KRT7** | 3.64E-03 | 1.64954 |
| **ADH1B** | 5.04E-03 | 1.64745 |
| **TGFBI** | 3.05E-04 | 1.63942 |
| **ACSL6** | 2.34E-03 | 1.62589 |
| **GIMAP8** | 4.35E-03 | 1.61832 |
| **NOL6** | 1.86E-02 | 1.61689 |
| **CAPS** | 8.21E-04 | 1.60325 |
| **NOL4** | 6.62E-05 | 1.59986 |
| **PTPRJ** | 1.73E-02 | 1.59955 |
| **C6** | 7.39E-03 | 1.59859 |
| **APOBR** | 6.30E-03 | 1.59834 |
| **APIP** | 5.99E-04 | 1.59571 |
| **FBN1** | 8.36E-03 | 1.59396 |
| **ACSL4** | 3.61E-04 | 1.59134 |
| **NCKAP1L** | 4.36E-03 | 1.58917 |
| **CD1E** | 1.56E-02 | 1.58042 |
| **COL6A2** | 1.60E-02 | 1.56789 |
| **CSDA, YBX3** | 2.62E-03 | 1.56347 |
| **ANXA3** | 4.22E-03 | 1.55952 |
| **PFKP** | 5.64E-04 | 1.55585 |
| **GLUL** | 6.48E-05 | 1.5524 |
| **C7** | 1.84E-03 | 1.55152 |
| **POMGNT1** | 9.46E-03 | 1.55077 |
| **MFAP5** | 4.95E-03 | 1.5465 |
| **MFAP2** | 2.96E-02 | 1.54539 |
| **IFITM2** | 3.13E-02 | 1.54518 |
| **SNCG** | 4.55E-02 | 1.54422 |
| **CASR** | 6.35E-03 | 1.54399 |
| **H2AFY2** | 2.32E-04 | 1.54336 |
| **TAP2** | 2.44E-02 | 1.54259 |
| **MBOAT7** | 3.84E-06 | 1.53754 |
| **GNG5** | 1.25E-02 | 1.53537 |
| **IGLV1-47** | 1.04E-02 | 1.53455 |
| **COL4A2** | 3.46E-03 | 1.52971 |
| **MARCKS** | 8.62E-03 | 1.52849 |
| **COL6A1** | 1.62E-02 | 1.52713 |
| **EFEMP1** | 9.46E-04 | 1.52477 |
| **DDX58** | 1.43E-04 | 1.52205 |
| **ABCA6** | 2.49E-04 | 1.52062 |
| **MNDA** | 2.67E-03 | 1.51874 |
| **TMEM72** | 3.54E-02 | 1.51861 |
| **MOXD1** | 2.18E-02 | 1.51491 |
| **PPP1R18** | 1.31E-02 | 1.51413 |
| **MUC1** | 2.13E-04 | 1.51285 |
| **PYCARD** | 6.78E-03 | 1.51179 |
| **CEP44** | 4.10E-04 | 1.50997 |
| **NPC1** | 4.27E-02 | 1.50696 |
| **PKN1** | 3.20E-05 | 1.5067 |
| **RAB25** | 1.65E-03 | 1.50419 |
| **RCC1** | 1.22E-06 | 1.50282 |
| **SLC12A1** | 2.34E-02 | 1.50164 |
| **MATN2** | 3.72E-03 | 1.50032 |
| **MVP** | 5.70E-04 | 1.49823 |
| **SCIN** | 7.10E-04 | 1.49742 |
| **TINAGL1** | 2.84E-02 | 1.49721 |
| **C5** | 1.94E-02 | 1.49236 |
| **MORF4L2** | 1.13E-02 | 1.49036 |
| **SEC24D** | 3.37E-05 | 1.48907 |
| **IGHV1-24** | 2.46E-02 | 1.48374 |
| **GNB4** | 1.72E-02 | 1.48163 |
| **MTHFR** | 4.25E-04 | 1.48053 |
| **RCN3** | 4.54E-02 | 1.47836 |
| **IGHV5-51** | 1.16E-02 | 1.478 |
| **SRSF10, LOC100996657** | 6.85E-03 | 1.47471 |
| **KHNYN** | 2.08E-03 | 1.47357 |
| **SP100** | 7.44E-04 | 1.46998 |
| **ATG5** | 4.66E-02 | 1.46636 |
| **VCAM1** | 4.35E-03 | 1.46621 |
| **RPL19** | 2.55E-02 | 1.46523 |
| **PGLYRP2** | 2.93E-02 | 1.4641 |
| **TMEM173** | 5.15E-03 | 1.46394 |
| **RWDD4** | 1.13E-03 | 1.46357 |
| **CORO1A** | 9.83E-05 | 1.46261 |
| **TANC2** | 2.15E-02 | 1.4589 |
| **GNAI1** | 5.68E-03 | 1.45792 |
| **TBC1D4** | 2.00E-03 | 1.45671 |
| **TMSB4X** | 3.25E-02 | 1.4564 |
| **COL3A1** | 4.52E-02 | 1.45639 |
| **PAPLN** | 2.17E-02 | 1.45603 |
| **AKAP13** | 2.23E-02 | 1.45601 |
| **MMP2** | 1.93E-02 | 1.45571 |
| **MYOF** | 9.13E-04 | 1.45545 |
| **FAM129A** | 5.45E-03 | 1.45294 |
| **IGKV1D-33** | 2.16E-02 | 1.4523 |
| **CD97, ADGRE5** | 6.08E-03 | 1.45104 |
| **MGP** | 1.74E-02 | 1.4483 |
| **PCNP** | 2.00E-02 | 1.44443 |
| **ABHD4** | 6.02E-03 | 1.44402 |
| **AOC3** | 3.40E-03 | 1.44297 |
| **KCNJ16** | 3.85E-03 | 1.4406 |
| **ATM** | 8.88E-05 | 1.44012 |
| **COL6A3** | 1.59E-02 | 1.43801 |
| **SOD3** | 3.25E-03 | 1.43655 |
| **TNFAIP2** | 1.06E-02 | 1.43563 |
| **OXNAD1** | 4.89E-03 | 1.4355 |
| **ACSS2** | 6.84E-05 | 1.43076 |
| **C8B** | 2.98E-02 | 1.42816 |
| **WDR72** | 2.35E-02 | 1.42811 |
| **FMO3** | 1.67E-02 | 1.42791 |
| **HTATIP2** | 3.93E-03 | 1.4272 |
| **IREB2** | 6.63E-03 | 1.42643 |
| **FBLN1** | 7.45E-03 | 1.42514 |
| **SETX** | 6.08E-03 | 1.42405 |
| **PDLIM7** | 3.02E-02 | 1.42201 |
| **FAAH** | 2.23E-03 | 1.4206 |
| **HS6ST1** | 2.91E-02 | 1.42044 |
| **BECN1** | 3.16E-03 | 1.41978 |
| **LONP2** | 1.89E-02 | 1.4176 |
| **RPAP1** | 5.98E-03 | 1.41732 |
| **GALNT10** | 1.94E-02 | 1.4171 |
| **CD163** | 3.30E-02 | 1.41685 |
| **TRIAP1** | 1.05E-03 | 1.41351 |
| **RPS27** | 1.53E-05 | 1.411 |
| **EMID1** | 2.32E-02 | 1.4085 |
| **WDR12** | 2.55E-04 | 1.40629 |
| **PLSCR1** | 1.11E-03 | 1.40497 |
| **HDAC4** | 2.83E-05 | 1.4045 |
| **COL15A1** | 1.79E-03 | 1.40426 |
| **FLAD1** | 8.57E-05 | 1.40279 |
| **C8G** | 1.92E-02 | 1.40195 |
| **KIDINS220** | 7.97E-03 | 1.39799 |
| **RCCD1** | 1.78E-02 | 1.39744 |
| **AKAP1** | 4.02E-02 | 1.39679 |
| **FOLR2** | 8.06E-04 | 1.39672 |
| **HDGFRP2, HDGFL2** | 8.75E-05 | 1.39618 |
| **DNAJC16** | 3.19E-03 | 1.39475 |
| **EMILIN1** | 3.25E-03 | 1.39209 |
| **PARP4** | 6.26E-04 | 1.39193 |
| **COL4A1** | 3.77E-02 | 1.39177 |
| **ITPA** | 2.16E-02 | 1.39109 |
| **LEMD2** | 4.67E-04 | 1.3905 |
| **COL4A3** | 1.27E-02 | 1.38925 |
| **CBFB** | 1.72E-03 | 1.38912 |
| **DDX21** | 2.20E-05 | 1.38811 |
| **AKAP12** | 3.86E-02 | 1.38661 |
| **BRK1** | 5.96E-04 | 1.3816 |
| **FRAS1** | 1.16E-02 | 1.38072 |
| **STAB1** | 4.96E-02 | 1.37965 |
| **ATP9B** | 4.27E-02 | 1.37812 |
| **PPIC** | 2.22E-02 | 1.37613 |
| **RRS1** | 2.18E-02 | 1.37573 |
| **RTF1** | 8.15E-03 | 1.37372 |
| **PARP14** | 5.47E-03 | 1.37359 |
| **RAPGEF1** | 2.06E-02 | 1.37354 |
| **FAF1** | 1.22E-07 | 1.37141 |
| **GGCX** | 2.77E-03 | 1.37057 |
| **PPID** | 2.63E-02 | 1.36811 |
| **CDH11** | 4.25E-04 | 1.36792 |
| **SOAT1** | 6.17E-04 | 1.36772 |
| **EML3** | 1.47E-02 | 1.36771 |
| **RRP12** | 3.66E-04 | 1.36582 |
| **CERS4** | 1.56E-02 | 1.36538 |
| **ABHD12** | 2.62E-02 | 1.36528 |
| **TES** | 4.85E-02 | 1.3649 |
| **OLFML3** | 1.48E-02 | 1.3638 |
| **THAP4** | 2.74E-02 | 1.36202 |
| **ASCC3** | 1.47E-02 | 1.36137 |
| **LAMA2** | 8.70E-03 | 1.36092 |
| **C7orf50** | 1.29E-02 | 1.35999 |
| **WDR89** | 2.91E-02 | 1.35949 |
| **DNAJB4** | 2.22E-02 | 1.35946 |
| **MICU1** | 1.16E-03 | 1.35934 |
| **METAP1D** | 1.49E-02 | 1.35906 |
| **LPCAT3** | 4.67E-03 | 1.35884 |
| **LMBRD2** | 2.76E-02 | 1.35813 |
| **PTMA** | 2.81E-02 | 1.35731 |
| **SMARCAD1** | 5.01E-03 | 1.35675 |
| **HEXB** | 3.27E-02 | 1.35449 |
| **CAP2** | 5.90E-04 | 1.35353 |
| **NELFCD, TH1L** | 6.61E-03 | 1.35266 |
| **HSPA13** | 1.28E-02 | 1.35246 |
| **KRT18** | 1.59E-03 | 1.35193 |
| **DEPTOR** | 5.32E-03 | 1.34913 |
| **COPG2** | 8.79E-04 | 1.34826 |
| **SLC27A3** | 1.38E-03 | 1.34817 |
| **PIK3CB** | 1.82E-02 | 1.34795 |
| **PIK3R2** | 2.91E-04 | 1.34736 |
| **BIRC6** | 2.41E-03 | 1.34574 |
| **TIMP1** | 4.23E-02 | 1.34549 |
| **PRDX4** | 7.90E-04 | 1.34543 |
| **SYVN1** | 5.71E-03 | 1.34295 |
| **GABARAPL2** | 1.64E-02 | 1.3383 |
| **TMX2** | 9.58E-03 | 1.33718 |
| **SCRN1** | 5.03E-03 | 1.33686 |
| **RAB23** | 2.90E-02 | 1.33641 |
| **IRF2BP2** | 3.74E-02 | 1.33548 |
| **LCP1** | 2.27E-03 | 1.33456 |
| **ENPP1** | 3.66E-05 | 1.33343 |
| **ALG8** | 8.14E-04 | 1.33302 |
| **MAT1A** | 4.66E-02 | 1.33192 |
| **MRPL10** | 1.02E-02 | 1.33038 |
| **TBCK** | 5.34E-03 | 1.33037 |
| **C11orf58** | 4.92E-03 | 1.3302 |
| **NUBP1** | 1.98E-03 | 1.32998 |
| **ATP1A2** | 2.66E-02 | 1.32984 |
| **THRAP3** | 7.59E-04 | 1.32959 |
| **MTA2** | 3.25E-05 | 1.32926 |
| **CSTB** | 2.74E-03 | 1.32779 |
| **ADAM17** | 4.02E-03 | 1.32773 |
| **TM9SF1** | 2.89E-05 | 1.32625 |
| **PITPNM1** | 4.11E-02 | 1.32618 |
| **DPF2** | 2.60E-03 | 1.32476 |
| **PELP1** | 1.81E-02 | 1.32436 |
| **SLC27A1** | 3.80E-03 | 1.32319 |
| **ATP13A1** | 1.11E-04 | 1.32299 |
| **TAOK3** | 1.58E-02 | 1.32295 |
| **NDUFAF6** | 9.10E-04 | 1.32217 |
| **BTAF1** | 3.42E-02 | 1.32202 |
| **DNM3** | 2.70E-03 | 1.32102 |
| **NUDT15** | 3.46E-02 | 1.32086 |
| **FLNB** | 5.64E-05 | 1.32046 |
| **ANO10** | 1.75E-03 | 1.32006 |
| **RNF31** | 7.07E-04 | 1.31946 |
| **RNF213** | 3.83E-02 | 1.31908 |
| **ROBO2** | 1.02E-02 | 1.31781 |
| **RPF2** | 1.56E-03 | 1.31668 |
| **MTMR12** | 9.65E-07 | 1.31648 |
| **IFI16** | 3.25E-03 | 1.31639 |
| **CTSD** | 1.76E-02 | 1.31429 |
| **RER1** | 1.49E-03 | 1.31396 |
| **CORO7** | 4.47E-06 | 1.31309 |
| **ANKRD17** | 1.25E-04 | 1.3123 |
| **CEBPZ** | 3.16E-04 | 1.31227 |
| **PIEZO1** | 2.78E-02 | 1.31222 |
| **GMPPB** | 7.58E-04 | 1.3122 |
| **NME7** | 4.59E-05 | 1.31169 |
| **U2SURP** | 3.72E-03 | 1.31142 |
| **RPL4** | 1.60E-02 | 1.31129 |
| **CHORDC1** | 1.26E-03 | 1.31115 |
| **RECQL** | 1.51E-03 | 1.31048 |
| **NR2F2** | 6.70E-03 | 1.31011 |
| **AGO2, EIF2C2** | 1.42E-02 | 1.30935 |
| **ARL6IP4** | 6.38E-03 | 1.30912 |
| **WWOX** | 1.60E-05 | 1.30736 |
| **PLCG1** | 4.82E-03 | 1.30594 |
| **CDH1** | 3.60E-03 | 1.30519 |
| **TRAPPC11** | 1.04E-02 | 1.30457 |
| **PAOX** | 3.67E-02 | 1.3045 |
| **BAD** | 2.66E-02 | 1.30448 |
| **DENND4C** | 9.52E-04 | 1.30278 |
| **LRRC59** | 4.18E-02 | 1.30277 |
| **PTRH2** | 1.95E-05 | 1.30197 |
| **NOP2** | 1.62E-02 | 1.30192 |
| **TRAF2** | 3.44E-02 | 1.30048 |
| **PSMG3** | 3.98E-02 | 1.2999 |
| **CEACAM1** | 1.82E-02 | 1.29967 |
| **SRP19** | 1.30E-02 | 1.29932 |
| **KCNJ15** | 4.72E-02 | 1.29929 |
| **MAP4K3** | 4.82E-02 | 1.2982 |
| **CTBP2** | 9.78E-03 | 1.298 |
| **NHP2L1, SNU13** | 1.31E-02 | 1.297 |
| **ATL3** | 3.12E-04 | 1.29485 |
| **RHOG** | 1.12E-02 | 1.29456 |
| **HEXA** | 2.31E-02 | 1.29443 |
| **CLCNKB** | 3.53E-02 | 1.29373 |
| **SREK1** | 3.07E-04 | 1.29362 |
| **FASN** | 2.22E-03 | 1.29353 |
| **PAK2** | 4.53E-02 | 1.29336 |
| **CD14** | 1.14E-03 | 1.29296 |
| **VPS8** | 8.49E-04 | 1.2929 |
| **RABL5, IFT22** | 8.77E-03 | 1.29278 |
| **SEMA3B** | 1.24E-02 | 1.29174 |
| **SCAMP4** | 4.08E-03 | 1.29157 |
| **CASP1** | 2.25E-03 | 1.29041 |
| **GOSR1** | 1.18E-02 | 1.29038 |
| **GBF1** | 1.33E-03 | 1.2902 |
| **TUBB4A** | 1.08E-02 | 1.28964 |
| **ABHD5** | 2.72E-02 | 1.28947 |
| **PACS2** | 1.21E-02 | 1.28939 |
| **XPC** | 1.52E-02 | 1.28911 |
| **OSCP1** | 3.77E-02 | 1.28886 |
| **XRN2** | 1.10E-07 | 1.28821 |
| **RHOT2** | 2.53E-02 | 1.28755 |
| **VMA21** | 1.19E-02 | 1.2873 |
| **COG2** | 3.60E-06 | 1.28654 |
| **DIP2B** | 4.25E-04 | 1.28654 |
| **CCS** | 2.58E-03 | 1.28651 |
| **STAT1** | 3.22E-03 | 1.28565 |
| **DIDO1** | 2.19E-03 | 1.28538 |
| **RBM28** | 6.54E-04 | 1.28476 |
| **NUFIP2** | 4.32E-02 | 1.28444 |
| **PRMT7** | 4.41E-02 | 1.28389 |
| **SAMHD1** | 3.14E-02 | 1.28233 |
| **UBR4** | 4.74E-04 | 1.28194 |
| **MICAL3** | 7.45E-03 | 1.28111 |
| **C17orf49, RNASEK** | 2.23E-02 | 1.28102 |
| **S100A11** | 1.40E-02 | 1.28047 |
| **GLRX2** | 1.20E-02 | 1.28039 |
| **FBLIM1** | 1.16E-02 | 1.27968 |
| **RBP1** | 2.08E-02 | 1.2792 |
| **VPS37A** | 3.18E-03 | 1.27867 |
| **USP19** | 1.37E-02 | 1.27804 |
| **DNAJA4** | 7.61E-03 | 1.27782 |
| **VWA5A** | 6.49E-03 | 1.27724 |
| **MINK1** | 2.95E-02 | 1.27717 |
| **ALG9** | 4.45E-02 | 1.27652 |
| **ARL1** | 3.69E-03 | 1.27635 |
| **INTS1** | 2.91E-02 | 1.27621 |
| **YBX1** | 2.74E-02 | 1.27591 |
| **PPME1** | 3.35E-04 | 1.27542 |
| **SQSTM1** | 9.98E-03 | 1.27533 |
| **STAT3** | 6.35E-04 | 1.27508 |
| **STMN1** | 1.30E-03 | 1.27436 |
| **FBXW8** | 3.07E-02 | 1.27414 |
| **NLN** | 6.59E-03 | 1.27248 |
| **GSPT1** | 1.51E-02 | 1.27012 |
| **SMPDL3B** | 1.93E-02 | 1.27004 |
| **AP1M2** | 2.93E-02 | 1.26973 |
| **VPS37B** | 3.06E-04 | 1.26919 |
| **MYO1D** | 1.92E-04 | 1.26847 |
| **WIPI2** | 1.74E-03 | 1.26834 |
| **RAB2B** | 1.11E-03 | 1.26802 |
| **LRP1** | 2.63E-02 | 1.26778 |
| **IAH1** | 7.16E-03 | 1.26718 |
| **ARHGEF6** | 8.46E-03 | 1.26671 |
| **POP1** | 6.01E-03 | 1.26568 |
| **ARMCX1** | 2.01E-02 | 1.26561 |
| **SERPINH1** | 9.85E-04 | 1.26557 |
| **GRN** | 1.58E-02 | 1.26514 |
| **CIAPIN1** | 4.26E-03 | 1.26492 |
| **S100A13** | 3.48E-02 | 1.26403 |
| **SDCBP** | 4.63E-02 | 1.26393 |
| **EPB41L2** | 4.03E-03 | 1.26317 |
| **ATAD1** | 1.94E-06 | 1.26186 |
| **HCFC1** | 3.21E-03 | 1.26095 |
| **TCEAL4** | 1.68E-02 | 1.26094 |
| **ANP32E** | 3.50E-03 | 1.26056 |
| **ZFPL1** | 2.63E-02 | 1.26034 |
| **KRT8** | 1.41E-02 | 1.26029 |
| **RBCK1** | 1.73E-02 | 1.25978 |
| **HTT** | 5.46E-03 | 1.25963 |
| **H2AFY** | 1.86E-02 | 1.25963 |
| **F8A2, F8A1, F8A3** | 3.47E-02 | 1.25905 |
| **PTDSS1** | 1.09E-02 | 1.25901 |
| **TMEM65** | 3.49E-03 | 1.25857 |
| **KIF1C** | 1.03E-02 | 1.258 |
| **WDR82** | 6.36E-03 | 1.25798 |
| **MAP2K1** | 1.95E-02 | 1.25734 |
| **PBXIP1** | 2.83E-03 | 1.25695 |
| **C9orf64** | 4.32E-05 | 1.25556 |
| **RBM39** | 1.19E-05 | 1.2554 |
| **RABEP2** | 3.41E-02 | 1.25522 |
| **RPS5** | 9.26E-04 | 1.25505 |
| **NAMPT** | 3.69E-03 | 1.25494 |
| **ALG2** | 3.52E-02 | 1.25428 |
| **ARHGEF17** | 1.36E-03 | 1.25395 |
| **SMARCA2** | 2.10E-02 | 1.25343 |
| **POGZ** | 3.96E-02 | 1.25288 |
| **AKAP9** | 1.31E-03 | 1.25139 |
| **ELMO2** | 5.09E-03 | 1.25063 |
| **MYH10** | 1.38E-03 | 1.2498 |
| **MARCKSL1** | 1.12E-02 | 1.24918 |
| **FUNDC2** | 2.43E-02 | 1.24891 |
| **GNG2** | 1.70E-02 | 1.24889 |
| **ZC3HAV1L** | 4.95E-02 | 1.24884 |
| **ANKRD13A** | 3.81E-03 | 1.24844 |
| **MCU** | 2.00E-03 | 1.24805 |
| **SEC63** | 4.93E-05 | 1.24779 |
| **ARMCX2** | 8.38E-03 | 1.24551 |
| **CAPN5** | 8.66E-04 | 1.24496 |
| **PLA2G16** | 3.82E-02 | 1.24451 |
| **FDXR** | 8.28E-03 | 1.24359 |
| **SEC11C** | 6.14E-03 | 1.24308 |
| **FUBP3** | 1.75E-02 | 1.24151 |
| **CLIP2** | 4.54E-02 | 1.24095 |
| **HIP1** | 1.37E-02 | 1.24084 |
| **ARMCX3** | 3.98E-03 | 1.24003 |
| **RFTN1** | 2.37E-03 | 1.23987 |
| **TMEM33** | 1.20E-02 | 1.23938 |
| **MTHFD1L** | 5.49E-03 | 1.23932 |
| **GFPT1** | 1.61E-03 | 1.23873 |
| **CD74** | 1.50E-02 | 1.23862 |
| **PSMB9** | 1.08E-02 | 1.23763 |
| **CUL7** | 1.24E-02 | 1.23758 |
| **COMMD10** | 3.85E-02 | 1.2372 |
| **BRIX1** | 3.67E-03 | 1.23702 |
| **ATG2A** | 3.10E-02 | 1.23625 |
| **KIFAP3** | 2.38E-02 | 1.23551 |
| **RBBP5** | 2.52E-02 | 1.23551 |
| **ATG7** | 2.06E-03 | 1.23543 |
| **CTNNBL1** | 4.78E-02 | 1.23516 |
| **IGFBP7** | 1.54E-02 | 1.23473 |
| **PLEC** | 4.88E-03 | 1.23459 |
| **SEH1L** | 2.57E-03 | 1.2339 |
| **TTC37** | 5.60E-04 | 1.23383 |
| **LDHA** | 1.10E-02 | 1.23346 |
| **PCM1** | 4.72E-03 | 1.23303 |
| **SLC33A1** | 4.98E-04 | 1.2326 |
| **TSTA3** | 1.26E-04 | 1.23256 |
| **KDELR1** | 4.35E-03 | 1.23228 |
| **LAS1L** | 9.82E-04 | 1.23219 |
| **RANBP2** | 1.07E-03 | 1.23208 |
| **SLC40A1** | 4.61E-02 | 1.23201 |
| **DYNLT1** | 1.92E-04 | 1.23169 |
| **AARS2** | 6.86E-03 | 1.23081 |
| **PIK3R4** | 6.86E-04 | 1.23075 |
| **CRELD1** | 1.14E-02 | 1.23069 |
| **CLCN3** | 4.68E-02 | 1.23058 |
| **CAD** | 3.05E-02 | 1.22901 |
| **SYMPK** | 9.51E-03 | 1.22889 |
| **TBL2** | 3.96E-04 | 1.22851 |
| **ATG14** | 1.26E-02 | 1.22844 |
| **ADAR** | 8.16E-04 | 1.2283 |
| **PTPLB, HACD2** | 1.01E-02 | 1.22811 |
| **ENGASE** | 9.06E-03 | 1.22802 |
| **NDUFAF1** | 1.04E-02 | 1.2279 |
| **LIMS1** | 1.65E-03 | 1.22781 |
| **ADCK4, COQ8B** | 1.12E-02 | 1.22763 |
| **TPM4** | 3.28E-02 | 1.22763 |
| **PRKDC** | 1.44E-05 | 1.22732 |
| **INTS3** | 3.76E-02 | 1.22674 |
| **MGLL** | 2.27E-02 | 1.22608 |
| **SUPT16H** | 1.15E-04 | 1.22552 |
| **JMJD6** | 3.44E-02 | 1.22509 |
| **MAN2B1** | 2.77E-02 | 1.2241 |
| **BCLAF1** | 4.16E-02 | 1.22409 |
| **SBF1** | 3.40E-02 | 1.2238 |
| **ZC3HC1** | 1.28E-03 | 1.22372 |
| **PELO** | 2.66E-04 | 1.22303 |
| **SBNO1** | 3.80E-02 | 1.22287 |
| **RAP1GDS1** | 5.06E-05 | 1.22267 |
| **MAGED2** | 9.25E-06 | 1.22217 |
| **OBSL1** | 1.82E-03 | 1.22205 |
| **LMNB1** | 3.59E-04 | 1.22166 |
| **SNRPA** | 2.25E-02 | 1.22137 |
| **NOP56** | 2.08E-03 | 1.2213 |
| **CASP8** | 1.16E-02 | 1.221 |
| **ARHGEF1** | 4.41E-02 | 1.22096 |
| **NUTF2** | 6.36E-05 | 1.22057 |
| **GET4** | 2.29E-02 | 1.22052 |
| **SF3B3** | 6.51E-03 | 1.22032 |
| **EIF4G3** | 1.06E-02 | 1.21988 |
| **SLC35B2** | 2.01E-02 | 1.21983 |
| **RNF20** | 4.45E-02 | 1.21974 |
| **RALGAPA2** | 2.08E-02 | 1.21957 |
| **TRAPPC8** | 8.94E-03 | 1.21776 |
| **EPCAM** | 1.83E-03 | 1.21718 |
| **GGT5** | 3.08E-02 | 1.21686 |
| **EPM2AIP1** | 4.10E-03 | 1.21676 |
| **FKBP9** | 2.86E-03 | 1.21616 |
| **DPYSL2** | 4.43E-03 | 1.21605 |
| **TBC1D15** | 3.39E-04 | 1.21529 |
| **SRP72** | 5.37E-05 | 1.21517 |
| **RAB34** | 1.20E-02 | 1.21472 |
| **LSM3** | 1.80E-02 | 1.21442 |
| **ALG11** | 3.48E-02 | 1.21354 |
| **TKT** | 3.53E-02 | 1.21318 |
| **MTMR6** | 1.09E-04 | 1.21259 |
| **HLA-A** | 3.18E-02 | 1.21258 |
| **RPS27L** | 2.87E-03 | 1.21249 |
| **CHD4** | 4.89E-03 | 1.2117 |
| **TANGO6, TMCO7** | 9.24E-03 | 1.2104 |
| **GPR89A, GPR89B** | 1.81E-02 | 1.20997 |
| **NRP1** | 2.83E-02 | 1.20995 |
| **NUP205** | 2.17E-02 | 1.20993 |
| **PPIH** | 3.42E-03 | 1.20987 |
| **RAVER1** | 3.51E-02 | 1.2092 |
| **PRKCI** | 1.64E-02 | 1.20903 |
| **MBNL1** | 3.99E-02 | 1.20889 |
| **MMS19** | 5.16E-06 | 1.20769 |
| **CAPG** | 2.15E-02 | 1.20763 |
| **ITGB4** | 3.06E-02 | 1.20722 |
| **MGST3** | 2.01E-02 | 1.20579 |
| **YWHAH** | 1.50E-02 | 1.20554 |
| **UBE2D1** | 4.53E-02 | 1.20542 |
| **TRIP12** | 2.01E-02 | 1.20496 |
| **RAB3GAP1** | 2.09E-04 | 1.20482 |
| **BMS1** | 2.12E-02 | 1.20468 |
| **ADPGK** | 1.40E-02 | 1.20436 |
| **DAD1** | 1.07E-03 | 1.20414 |
| **SRPK1** | 1.48E-02 | 1.20362 |
| **TMEM147** | 1.69E-02 | 1.20348 |
| **RBMX** | 3.77E-02 | 1.20273 |
| **GDPGP1** | 1.27E-02 | 1.20235 |
| **APMAP** | 3.68E-03 | 1.20213 |
| **CLCC1** | 1.01E-03 | 1.20202 |
| **DNAJB1** | 9.75E-03 | 1.20164 |
| **UBXN4** | 1.81E-03 | 1.20156 |
| **RPA1** | 1.21E-04 | 1.20153 |
| **CHMP4A** | 6.29E-03 | 1.20127 |
| **INPPL1** | 4.51E-02 | 1.2005 |
| **ALG1** | 1.45E-02 | 1.20036 |
| **PLA2G15** | 1.66E-02 | 0.83271 |
| **RRAS** | 4.14E-02 | 0.83246 |
| **COX4I1** | 1.08E-02 | 0.83241 |
| **IDH3B** | 7.50E-04 | 0.8311 |
| **MRPL21** | 4.55E-03 | 0.83081 |
| **SAMM50** | 2.11E-03 | 0.8267 |
| **LASP1** | 4.94E-02 | 0.82641 |
| **PMPCA** | 1.31E-03 | 0.82622 |
| **MNF1, UQCC2** | 2.57E-03 | 0.82235 |
| **GNB1** | 2.57E-02 | 0.822 |
| **EZR** | 1.91E-02 | 0.8197 |
| **MDH2** | 1.39E-02 | 0.81885 |
| **UQCRC1** | 5.49E-03 | 0.81785 |
| **AMIGO1** | 4.08E-02 | 0.81751 |
| **SH3BGRL2** | 1.19E-02 | 0.81651 |
| **COX5A** | 1.27E-02 | 0.81624 |
| **SMPD1** | 4.66E-02 | 0.81607 |
| **ANG** | 4.67E-02 | 0.81587 |
| **ARHGEF15** | 5.01E-03 | 0.81456 |
| **SH3GL2** | 3.30E-02 | 0.81453 |
| **EARS2** | 7.37E-03 | 0.81266 |
| **CA2** | 4.08E-03 | 0.81224 |
| **BNIP3** | 8.60E-03 | 0.81083 |
| **IMMT** | 5.74E-03 | 0.8103 |
| **MRPL30** | 5.08E-03 | 0.81002 |
| **SH3BGRL3** | 2.41E-02 | 0.80987 |
| **ATP5B** | 4.16E-03 | 0.80946 |
| **RNF113A** | 3.69E-02 | 0.80945 |
| **NDUFA7** | 2.52E-02 | 0.80573 |
| **GPX4** | 5.60E-03 | 0.80567 |
| **RMND1** | 7.79E-03 | 0.80545 |
| **FASTKD5** | 2.50E-02 | 0.80409 |
| **IDH3A** | 1.16E-03 | 0.80263 |
| **PLA2R1** | 2.38E-02 | 0.80198 |
| **NPL** | 7.99E-03 | 0.80181 |
| **CNBP** | 3.78E-02 | 0.80126 |
| **COX7A2** | 1.29E-03 | 0.80022 |
| **ITGA3** | 2.81E-02 | 0.79923 |
| **C9orf89, CARD19** | 1.03E-02 | 0.79865 |
| **GPD1L** | 1.25E-02 | 0.79859 |
| **ATP5EP2** | 2.23E-02 | 0.79827 |
| **CLTB** | 7.37E-03 | 0.79817 |
| **ATPIF1** | 4.77E-02 | 0.79741 |
| **ATPAF2** | 3.37E-03 | 0.79688 |
| **NDUFA11** | 7.33E-04 | 0.79553 |
| **RAB15** | 2.12E-05 | 0.79523 |
| **FARP2** | 5.11E-03 | 0.79393 |
| **SPATS2L** | 4.16E-02 | 0.79179 |
| **TIMM21** | 2.47E-03 | 0.79039 |
| **ECHDC2** | 2.31E-03 | 0.79029 |
| **MRPS23** | 1.21E-02 | 0.79028 |
| **ATP6V1D** | 2.98E-02 | 0.78914 |
| **ATP6V1E1** | 9.21E-03 | 0.78773 |
| **SNX30** | 1.70E-02 | 0.78576 |
| **PTRH1** | 2.95E-03 | 0.78256 |
| **C17orf89, NDUFAF8** | 1.26E-02 | 0.7823 |
| **C3orf33** | 1.99E-04 | 0.78195 |
| **ACADM** | 4.80E-02 | 0.78184 |
| **CHL1** | 5.62E-03 | 0.78182 |
| **SLC25A17** | 1.45E-02 | 0.77968 |
| **CYB5A** | 1.86E-02 | 0.77843 |
| **ACTR1B** | 3.90E-04 | 0.77817 |
| **PRNP** | 1.82E-03 | 0.77556 |
| **CHMP2B** | 7.08E-04 | 0.77462 |
| **NDUFB7** | 2.29E-03 | 0.77405 |
| **GCSH** | 4.13E-02 | 0.77275 |
| **NDUFA13** | 1.51E-04 | 0.77246 |
| **TPP1** | 1.31E-02 | 0.77128 |
| **ATP6V1C1** | 2.87E-02 | 0.77092 |
| **ATP6AP1** | 1.39E-02 | 0.7702 |
| **CBR4** | 1.04E-03 | 0.76978 |
| **ETHE1** | 6.34E-03 | 0.76969 |
| **HNRNPAB** | 2.57E-02 | 0.76777 |
| **COQ3** | 2.19E-03 | 0.7665 |
| **IGKV2-40, IGKV2D-40** | 4.83E-02 | 0.76595 |
| **UQCRQ** | 7.74E-04 | 0.76581 |
| **AZGP1** | 2.42E-02 | 0.7642 |
| **CWC15** | 3.02E-02 | 0.76254 |
| **CMC1** | 6.40E-05 | 0.76196 |
| **GLYATL1** | 3.87E-02 | 0.76122 |
| **CLTA** | 1.09E-02 | 0.76015 |
| **MPST** | 2.25E-02 | 0.75611 |
| **ATP6AP2** | 7.44E-03 | 0.75528 |
| **SERPINF1** | 3.35E-02 | 0.75515 |
| **GLUD1** | 3.66E-02 | 0.75359 |
| **MRPL54** | 3.33E-03 | 0.75349 |
| **UBR5** | 2.47E-02 | 0.75327 |
| **COX6B1** | 7.30E-04 | 0.75211 |
| **ITGA2B** | 3.75E-02 | 0.75147 |
| **ACADSB** | 4.21E-02 | 0.75142 |
| **MACROD1** | 3.58E-02 | 0.75084 |
| **CALML4** | 2.30E-02 | 0.75053 |
| **COL4A4** | 4.55E-03 | 0.75037 |
| **NDUFB5** | 8.79E-05 | 0.74939 |
| **SLC9A3R2** | 1.17E-03 | 0.7474 |
| **THY1** | 3.65E-02 | 0.74559 |
| **C19orf52, TIMM29** | 3.15E-07 | 0.74498 |
| **BCKDK** | 2.88E-03 | 0.74356 |
| **AADAT** | 4.39E-02 | 0.73754 |
| **TMEM70** | 2.37E-03 | 0.7369 |
| **NMRAL1** | 1.96E-02 | 0.73401 |
| **SLC51B** | 4.25E-02 | 0.73325 |
| **AIF1** | 4.88E-05 | 0.7314 |
| **NDUFB4** | 1.88E-03 | 0.72864 |
| **NDUFC2, NDUFC2, KCTD14** | 2.21E-04 | 0.72862 |
| **UBAC2** | 1.38E-02 | 0.72858 |
| **CADM4** | 5.32E-04 | 0.72803 |
| **PARM1** | 2.64E-03 | 0.72751 |
| **COX7A2L** | 4.88E-03 | 0.72718 |
| **SDC1** | 4.60E-02 | 0.72707 |
| **VAMP3** | 3.65E-03 | 0.72427 |
| **CYSTM1** | 7.98E-03 | 0.72313 |
| **PRODH** | 7.44E-03 | 0.72114 |
| **HDHD3** | 5.84E-03 | 0.72092 |
| **NPHS1** | 6.65E-03 | 0.71897 |
| **BPGM** | 3.77E-02 | 0.71781 |
| **REPS2** | 3.19E-02 | 0.71738 |
| **IDH3G** | 5.35E-07 | 0.71726 |
| **MAPT** | 3.60E-02 | 0.71662 |
| **NES** | 6.54E-04 | 0.71457 |
| **APOH** | 3.85E-02 | 0.71433 |
| **FOLR1** | 1.95E-02 | 0.71374 |
| **APOO** | 7.91E-05 | 0.71229 |
| **GALK1** | 3.65E-03 | 0.71219 |
| **MTRF1L** | 3.99E-02 | 0.70805 |
| **C1orf123** | 2.68E-03 | 0.70714 |
| **NRIP2** | 1.10E-02 | 0.70428 |
| **CLYBL** | 1.66E-03 | 0.70394 |
| **AGXT2L2, PHYKPL** | 3.82E-03 | 0.69897 |
| **TXNRD2** | 2.76E-04 | 0.69825 |
| **PHGDH** | 5.15E-03 | 0.69795 |
| **GOLPH3L** | 6.63E-04 | 0.69715 |
| **SLCO4C1** | 6.01E-03 | 0.69537 |
| **SYNPO** | 5.19E-05 | 0.69524 |
| **TTR** | 1.04E-02 | 0.6943 |
| **VAMP2** | 1.66E-04 | 0.69341 |
| **GLUD2** | 3.34E-05 | 0.69219 |
| **IGKV1-17** | 4.70E-03 | 0.68959 |
| **FAM151A** | 2.00E-02 | 0.68787 |
| **IGHV3-74** | 4.15E-02 | 0.6805 |
| **ALPL** | 7.57E-03 | 0.67227 |
| **THTPA** | 1.15E-04 | 0.66923 |
| **CLIC5** | 4.11E-03 | 0.66292 |
| **TUBB4B** | 5.93E-03 | 0.66211 |
| **MAGI2** | 1.09E-02 | 0.66072 |
| **AKAP10** | 2.37E-04 | 0.66044 |
| **SLC34A3** | 8.20E-03 | 0.66008 |
| **CDH13** | 4.75E-02 | 0.65703 |
| **NDUFA2** | 5.88E-05 | 0.65627 |
| **PDLIM2** | 6.86E-04 | 0.65473 |
| **GCDH** | 3.69E-02 | 0.65457 |
| **CLDN2** | 2.74E-03 | 0.65412 |
| **GYS1** | 7.11E-04 | 0.64743 |
| **EMCN** | 7.79E-03 | 0.64372 |
| **APOC3** | 2.76E-02 | 0.64265 |
| **VMP1** | 4.89E-03 | 0.64238 |
| **C1S** | 1.74E-02 | 0.63988 |
| **NPHS2** | 7.88E-04 | 0.63458 |
| **GSTA1** | 2.53E-02 | 0.63451 |
| **CLEC18A** | 4.35E-02 | 0.62807 |
| **ACTN2** | 2.74E-04 | 0.62304 |
| **IGKV2-30** | 3.28E-02 | 0.62282 |
| **ABP1, AOC1** | 2.25E-02 | 0.62198 |
| **ERP27** | 2.72E-02 | 0.61411 |
| **DSC1** | 3.68E-02 | 0.60943 |
| **RBP4** | 1.59E-02 | 0.60151 |
| **FGF1** | 2.53E-02 | 0.59947 |
| **PODXL** | 2.53E-04 | 0.58711 |
| **FTL** | 2.90E-02 | 0.57745 |
| **DDN** | 1.80E-02 | 0.57517 |
| **TBC1D2B** | 1.98E-03 | 0.57276 |
| **KRT6B** | 2.24E-02 | 0.56946 |
| **DPEP1** | 2.97E-03 | 0.56884 |
| **EHD3** | 2.40E-06 | 0.56125 |
| **NEBL** | 3.56E-04 | 0.5587 |
| **DPYD** | 4.07E-02 | 0.55226 |
| **PTPRO** | 1.14E-03 | 0.55185 |
| **ARSF** | 6.46E-03 | 0.54995 |
| **KRT1** | 2.33E-02 | 0.54463 |
| **CPNE6** | 1.13E-02 | 0.53245 |
| **CIRBP** | 3.75E-03 | 0.52733 |
| **YIPF5** | 5.21E-03 | 0.52569 |
| **CREBBP** | 1.52E-02 | 0.50977 |
| **PLSCR4** | 2.58E-05 | 0.49734 |
| **FTH1** | 5.33E-03 | 0.48907 |
| **CD99** | 2.61E-04 | 0.47571 |
| **KRT9** | 3.04E-02 | 0.47344 |
| **DHRS4L2** | 9.88E-04 | 0.45406 |
| **CALML3** | 4.62E-03 | 0.427 |

AKI: acute kidney injury
