# Supplementary material for: Proteomic analysis of human kidney biopsies unveils emerging acute kidney injury very early after liver graft reperfusion
Source: J Transl Med. 2025 Jun 16;23:658. doi: 10.1186/s12967-025-06695-w (PMC12172208; doi:10.1186/s12967-025-06695-w)
Supplement: Supplementary file 2 — Suplementary material 2. Table 2. Top 100 most up- and downregulated proteins in the AKI vs no AKI groups, comparison before transplantation. AKI, acute kidney injury; FC, fold change [file 12967_2025_6695_MOESM2_ESM.docx]

**Supplementary table 2**. Top 100 most up-, and downregulated proteins in the AKI 2/3 vs no AKI groups, comparison before transplantation

| UPREG. | |  |  |  |  |
| --- | --- | --- | --- | --- | --- |
| Symbol | **Name** | **P-value** | **FC** | **Biological function** | **Biological process** |
| TPSAB1 | tryptase alpha/beta 1 | 6.50E-06 | 7.29 | serine protease | serine-type peptidase activity |
| MMP7 | matrix metallopeptidase 7 | 4.57E-04 | 4.85 | metalloprotease | metalloendopeptidase activity |
| IGJ | joining chain of multimeric IgA and IgM | 1.72E-03 | 3.29 | immunoglobulin | antigen binding |
| FAM21C | Family With Sequence Similarity 21 Member C / WASH complex subunit 2C | 1.91E-02 | 3.03 | scaffold/adaptor protein | actin polymerization |
| MUC13 | mucin 13, cell surface associated | 2.14E-03 | 3 | homodimerization activity | cell signaling |
| IGLC7 | immunoglobulin lambda constant 7 | 8.51E-04 | 2.99 | immunoglobulin | antigen binding |
| MYCBP | MYC binding protein | 1.20E-04 | 2.83 | transcription cofactor | transcription coactivator activity |
| HSPB6 | heat shock protein family B (small) member 6 | 2.18E-03 | 2.65 | chaperone | protein folding chaperone |
| SLC39A9 | solute carrier family 39 member 9 | 4.82E-03 | 2.64 | secondary carrier transporter | zinc efflux transmembrane transporter activity |
| PROM1 | prominin 1 | 1.70E-02 | 2.58 | actinin binding | enables actinin binding |
| IGHV5-51 | immunoglobulin heavy variable 5-51 | 5.73E-07 | 2.54 | immunoglobulin | antigen binding |
| LTBP2 | latent transforming growth factor beta binding protein 3 | 1.55E-04 | 2.47 | extracellular matrix structural protein | growth factor binding |
| SFT2D3 | SFT2 domain containing 3 | 4.48E-03 | 2.39 | vesicle-mediated transport (predicted) | vesicle-mediated transport (predicted) |
| CMA1 | chymase 1 | 5.19E-04 | 2.33 | serine protease | endopeptidase activity |
| IGLV1-47 | immunoglobulin lambda variable 1-47 | 1.47E-05 | 2.31 | immunoglobulin | antigen binding |
| DES | desmin | 4.22E-02 | 2.27 | chaperone | cytoskeletal protein binding |
| KRT14 | keratin 14 | 3.37E-03 | 2.25 | intermediate filament | structural constituent of cytoskeleton |
| PTGIS | prostaglandin I2 synthase | 8.03E-04 | 2.24 | prostacyclin synthase | prostaglandin-I synthase activity |
| IGLV3-25 | immunoglobulin lambda variable 3-25 | 1.80E-02 | 2.23 | immunoglobulin | antigen binding |
| CPA3 | carboxypeptidase A4 | 1.29E-03 | 2.17 | metalloprotease | enables metallocarboxypeptidase activity |
| NQO1 | NAD(P)H quinone dehydrogenase 1 | 9.33E-03 | 2.05 | oxidoreductase | NADH dehydrogenase (quinone) activity |
| PDLIM3 | PDZ and LIM domain 3 | 9.92E-03 | 2.04 | actin or actin-binding cytoskeletal protein | actin binding |
| FBLN5 | fibulin 5 | 2.68E-04 | 2.01 | extracellular matrix structural protein | integrin binding |
| IGHV1-46 | immunoglobulin heavy variable 1-46 | 1.18E-04 | 2.01 | immunoglobulin | antigen binding |
| CRYAB | crystallin alpha B | 2.88E-03 | 2 | metalloprotease | microtubule binding |
| IGHV4-34 | immunoglobulin heavy variable 4-34 | 3.60E-03 | 1.97 | immunoglobulin | antigen binding |
| MZB1 | marginal zone B and B1 cell specific protein | 4.41E-03 | 1.92 | protein binding | enables protein binding |
| CES1 | carboxylesterase 1 | 1.61E-03 | 1.91 | esterase | carboxylic ester hydrolase activity |
| IGKV1-6 | immunoglobulin kappa variable 1-6 | 8.02E-04 | 1.91 | immunoglobulin | antigen binding |
| CES1P1 | carboxylesterase 1 pseudogene 1 | 1.86E-02 | 1.91 | esterase | carboxylic ester hydrolase activity |
| IGLV2-23 | immunoglobulin lambda variable 2-23 | 3.48E-04 | 1.91 | immunoglobulin | antigen binding |
| ITGB6 | integrin subunit beta 6 | 5.14E-04 | 1.9 | integrin | integrin binding |
| MFAP4 | microfibril associated protein 4 | 3.56E-02 | 1.88 | intercellular signal molecule | cell adhesion |
| MANBAL | mannosidase beta like | 3.42E-03 | 1.88 | mannose hydrolase | enables protein binding |
| LMOD1 | leiomodin 1 | 1.15E-02 | 1.86 | actin or actin-binding cytoskeletal protein | actin binding |
| CFHR1 | complement factor H related 1 | 1.26E-03 | 1.86 | complement component | complement component C3b binding |
| TGM2 | transglutaminase 2 | 3.66E-04 | 1.86 | transferase | protein-glutamine gamma-glutamyltransferase activity |
| PPP4R2 | protein phosphatase 4 regulatory subunit 2 | 1.08E-04 | 1.85 | phosphatase | protein phosphatase regulator activity |
| STAB1 | stabilin 1 | 1.43E-06 | 1.84 | membrane trafficking regulatory protein | scavenger receptor activity |
| TNXB | tenascin XB | 5.12E-04 | 1.8 | extracellular matrix protein | collagen binding |
| IGHM | immunoglobulin heavy constant mu | 2.24E-02 | 1.79 | immunoglobulin receptor superfamily | antigen binding |
| IGLV3-19 | immunoglobulin lambda variable 3-19 | 1.91E-03 | 1.79 | immunoglobulin | antigen binding |
| MFGE8 | milk fat globule EGF and factor V/VIII domain containing | 8.69E-04 | 1.79 | oxidoreductase | integrin binding |
| IGHV1-24 | immunoglobulin heavy variable 1-24 | 5.48E-04 | 1.78 | immunoglobulin | antigen binding |
| LSP1 | lymphocyte specific protein 1 | 6.49E-05 | 1.78 | non-motor actin binding protein | actin binding |
| RNASE2 | ribonuclease A family member 2 | 1.28E-02 | 1.77 | endoribonuclease | lyase activity |
| IGKV1D-33 | immunoglobulin kappa variable 1D-33 | 2.80E-03 | 1.77 | immunoglobulin | antigen binding |
| IGLV4-69 | immunoglobulin lambda variable 4-69 | 1.13E-04 | 1.76 | immunoglobulin | antigen binding |
| PLTP | phospholipid transfer protein | 1.55E-04 | 1.75 | defense/immunity protein | high- and lo-density lipoprotein particle binding |
| EMID1 | EMI domain containing 1 | 1.26E-03 | 1.75 | intermediate filament | protein binding |

| DOWNREG. | |  |  |  |  |
| --- | --- | --- | --- | --- | --- |
| Symbol | **Name** | **P-value** | **FC** | **Biological function** | **Biological process** |
| H2AFX | H2A variant histone | 7.18E-03 | 0.37 | histone | structural constituent of chromatin |
| CIRBP | cold inducible RNA binding protein | 2.29E-05 | 0.41 | RNA metabolism protein | translation repressor activity |
| FTH1 | ferritin heavy chain 1 | 4.20E-03 | 0.42 | storage protein | ferroxidase activity |
| AGXT | alanine--glyoxylate aminotransferase | 2.48E-03 | 0.46 | transaminase | alanine-glyoxylate transaminase activity |
| FTL | ferritin light chain | 1.27E-02 | 0.47 | storage protein | iron ion binding |
| RBP4 | RAB GTPase activating protein 1 like | 2.34E-02 | 0.49 | transfer/carrier protein | GTPase activator activity |
| MB | myoglobin | 2.33E-02 | 0.5 | globin | heme binding |
| DHRS4L2 | dehydrogenase/reductase 4 like 2 | 1.29E-02 | 0.51 | dehydrogenase | carbonyl reductase (NADPH) activity |
| ARSF | arylsulfatase F | 2.20E-03 | 0.51 | hydrolase | arylsulfatase activity |
| TUBB2B | tubulin beta 2B class IIb | 3.40E-03 | 0.53 | tubulin | structural constituent of cytoskeleton |
| TBC1D2B | TBC1 domain family member 2B | 2.47E-03 | 0.55 | GTPase-activating protein | GTPase activator activity |
| GCDH | glutaryl-CoA dehydrogenase | 1.32E-03 | 0.56 | dehydrogenase | glutaryl-CoA dehydrogenase activity |
| IPO4 | importin 4 | 7.20E-03 | 0.57 | transporter | nuclear import signal receptor activity |
| PLSCR4 | phospholipid scramblase 4 | 4.38E-05 | 0.57 | transporter | phospholipid scramblase activity |
| CCL14 | C-C motif chemokine ligand 14 | 2.85E-02 | 0.57 | cytokine | chemokine activity |
| ABP1 | amine oxidase copper containing 1 | 3.70E-02 | 0.57 | oxidase | degradation of Histamine |
| FAU | FAU ubiquitin like and ribosomal protein S30 fusion | 5.77E-03 | 0.57 | component of ribosome | RNA binding |
| AGT | alanine--glyoxylate aminotransferase | 2.84E-02 | 0.58 | protease inhibitor | growth factor activity |
| RPL29 | ribosomal protein L29 | 1.32E-02 | 0.58 | ribosomal protein | RNA binding - RNA translation |
| DPYD | dihydropyrimidine dehydrogenase | 4.84E-02 | 0.58 | dehydrogenase | dihydropyrimidine dehydrogenase (NADP+) activity |
| PDZK1IP1 | PDZK1 interacting protein 1 | 1.30E-02 | 0.58 | --- | --- |
| HBG1 | hemoglobin subunit gamma 1 | 2.91E-02 | 0.6 | globin | heme binding |
| YIPF5 | Yip1 domain family member 5 | 1.40E-05 | 0.6 | structural protein | endoplasmatic reticulum-Golgi transport |
| SLC34A3 | solute carrier family 34 member 3 | 1.98E-02 | 0.61 | secondary carrier transporter | sodium:phosphate symporter activity |
| AOX1 | aldehyde oxidase 1 | 2.33E-02 | 0.62 | oxidoreductase | oxidoreductase activity |
| CLYBL | citramalyl-CoA lyase | 1.44E-05 | 0.62 | lyase | hydrolase activity |
| NOLC1 | nucleolar and coiled-body phosphoprotein 1 | 7.99E-04 | 0.62 | nucleotide binding | regulator of RNA polymerase I |
| PHGDH | phosphoglycerate dehydrogenase | 4.25E-04 | 0.62 | dehydrogenase | electron transfer activity |
| NEBL | nebulette | 2.01E-04 | 0.63 | actin binding | actin filament binding |
| CD99 | CD99 molecule (Xg blood group) | 4.80E-03 | 0.64 | cell-cell adhesion | cell adhesion |
| COL6A6 | collagen type VI alpha 6 chain | 2.99E-02 | 0.64 | cell-binding protein | extracellular matrix component |
| SLC5A10 | solute carrier family 5 member 10 | 1.96E-02 | 0.64 | secondary carrier transporter | glucose:sodium symporter activity |
| NDUFV3 | NADH:ubiquinone oxidoreductase subunit V3 | 4.87E-03 | 0.64 | oxidoreductase | NADH dehydrogenase (ubiquinone) activity |
| WDR72 | WD repeat domain 72 | 4.94E-02 | 0.67 | --- | --- |
| SLC7A9 | solute carrier family 7 member 9 | 2.57E-03 | 0.67 | transporter | neutral amino acid transmembrane transporter activity |
| MAPT | microtubule associated protein tau | 1.30E-02 | 0.68 | actin binding | actin binding |
| TUBB4B | tubulin beta 4B class IVb | 3.06E-02 | 0.68 | tubulin | structural constituent of cytoskeleton |
| NEK7 | NIMA related kinase 7 | 2.20E-05 | 0.68 | non-receptor serine/threonine protein kinase | protein serine kinase activity |
| SRR | serine racemase | 1.91E-02 | 0.68 | epimerase/racemase | D-serine ammonia-lyase activity |
| NDUFA7 | NADH:ubiquinone oxidoreductase subunit A7 | 6.79E-06 | 0.69 | oxidoreductase | NADH dehydrogenase (ubiquinone) activity |
| GOLPH3L | golgi phosphoprotein 3 like | 3.62E-04 | 0.69 | lipid binding | phosphatidylinositol-4-phosphate binding |
| C5orf24 | chromosome 5 open reading frame 24 | 6.31E-03 | 0.69 | --- | --- |
| REPS2 | RALBP1 associated Eps domain containing 2 | 5.67E-03 | 0.69 | membrane traffic protein | growth factor receptors endocytosis |
| RABGAP1L | RAB GTPase Activating Protein 1 Like | 2.55E-02 | 0.69 | GTPase-activating protein | endocytosis and intracellular protein transport |
| GPX4 | glutathione peroxidase 4 | 4.74E-07 | 0.69 | peroxidase | glutathione peroxidase activity |
| SLC51B | solute carrier family 51 subunit beta | 1.48E-02 | 0.69 | transporter | bile acid transmembrane transporter activity |
| FAM98A | family with sequence similarity 98 member A | 2.06E-03 | 0.69 | RNA splicing factor | RNA binding |
| SLC13A2 | solute carrier family 13 member 2 | 5.24E-03 | 0.7 | secondary carrier transporter | alpha-ketoglutarate transmembrane transporter activity |
| COX7A2L | cytochrome c oxidase subunit 7A2 like | 6.92E-04 | 0.7 | oxidase | cytochrome-c oxidase activity |
| OCLN | occludin | 2.23E-02 | 0.7 | formation and regulation of the tight junction | cell adhesion |

FC: fold change
