# Supplementary material for: Proteomic analysis of human kidney biopsies unveils emerging acute kidney injury very early after liver graft reperfusion
Source: J Transl Med. 2025 Jun 16;23:658. doi: 10.1186/s12967-025-06695-w (PMC12172208; doi:10.1186/s12967-025-06695-w)
Supplement: Supplementary file 3 — Suplementary material 3. Table 3. Most differentially regulated pathways in the AKI vs no AKI groups, comparison before transplantation. AKI, acute kidney injury; FDR, false discovery rate [file 12967_2025_6695_MOESM3_ESM.docx]

**Supplementary table 3.** All regulated proteins in the AKI 2/3 group, before vs after transplantation

| **UPREGULATED PROTEIN** | **Fold change** |  | **DOWNREGULATED PROTEIN** | **Fold change** |
| --- | --- | --- | --- | --- |
| ADH1A | 8.281488172 |  | PTGIS | 0.551518058 |
| ADH4 | 8.137523344 |  | IGHV4-34 | 0.560516952 |
| AKR1C2 | 3.133097797 |  | SNRPF | 0.560594743 |
| SULT2A1 | 2.968223787 |  | COL3A1 | 0.579990029 |
| NNMT | 2.868585914 |  | LTBP2 | 0.588359538 |
| AKR1D1 | 2.84956596 |  | FBLN5 | 0.595593503 |
| SAA1 | 2.698055549 |  | IGHV3-43D | 0.596899463 |
| SLC5A1 | 2.295963805 |  | IGKV1-6 | 0.601080427 |
| SPP1 | 2.290178552 |  | IGHV5-51 | 0.604238502 |
| MMP9 | 2.131412733 |  | SERPINA6 | 0.607998596 |
| SLC5A8 | 2.057339759 |  | ECM1 | 0.608576364 |
| COL6A6 | 1.949859221 |  | IGKV2-30 | 0.612223408 |
| GDF15 | 1.848788243 |  | COL1A2 | 0.617709322 |
| NOLC1 | 1.822640358 |  | FMOD | 0.637510061 |
| PADI4 | 1.820670619 |  | IGHV3-7 | 0.6386512 |
| HIST1H1E | 1.762349566 |  | F10 | 0.642313659 |
| DHRS4L2 | 1.738845914 |  | COL1A1 | 0.642914978 |
| HBG1 | 1.728846907 |  | IGHV1-46 | 0.647350935 |
| TMSB4X | 1.719798873 |  | IGKV2-40 | 0.650296643 |
| SEC14L2 | 1.715429549 |  | SERPINA7 | 0.653201537 |
| AZU1 | 1.686333544 |  | IGKV3-15 | 0.653459636 |
| UCHL1 | 1.68097716 |  | KRT14 | 0.659287677 |
| AGXT | 1.652671501 |  | IGLV7-46 | 0.673621412 |
| TNFSF13 | 1.649295514 |  | IGLV3-25 | 0.676564863 |
| SLC7A5 | 1.640436893 |  | OGN | 0.677597195 |
| SLC12A1 | 1.640345739 |  | IGKV4-1 | 0.680845774 |
| MAP3K5 | 1.626408836 |  | IGLV1-47 | 0.683583659 |
| DNAJC12 | 1.624887655 |  | LRG1 | 0.684679201 |
| LTF | 1.616081477 |  | IGHV1-24 | 0.688367136 |
| HIST1H1B | 1.553303683 |  | EFEMP1 | 0.68977603 |
| TRIAP1 | 1.55233508 |  | IGKV3D-20 | 0.694260907 |
| MPO | 1.552066188 |  | IGKC | 0.695103558 |
| FAU | 1.538751231 |  | IGLV3-19 | 0.7000112 |
| CASR | 1.525888809 |  | HRG | 0.705588994 |
| OXCT1 | 1.525518987 |  | POSTN | 0.706469882 |
| PIK3AP1 | 1.524060307 |  | IGLV4-69 | 0.70852464 |
| ELANE | 1.501641039 |  | OLFML1 | 0.708716226 |
| S100A8 | 1.493399454 |  | GALNT10 | 0.711196438 |
| AQP2 | 1.492871708 |  | IGHV3-74 | 0.715721361 |
| PCNP | 1.483279152 |  | CRTAC1 | 0.718699208 |
| YBX1 | 1.466614671 |  | IGKV3-20 | 0.718928429 |
| MAT1A | 1.459212544 |  | REG1A | 0.720779529 |
| PDK4 | 1.448319563 |  | IGKV3D-11 | 0.723221711 |
| GPT | 1.427904925 |  | CD5L | 0.726180539 |
| WDR72 | 1.427063865 |  | AHSG | 0.728600564 |
| SLC1A5 | 1.423912226 |  | CTPS1 | 0.738121388 |
| NOL6 | 1.423695224 |  | ZNF704 | 0.738720285 |
| ARHGEF28 | 1.420776647 |  | IGKV1-17 | 0.744152376 |
| ABHD12 | 1.418267835 |  | COL12A1 | 0.746544368 |
| CTSL | 1.415890854 |  | COL4A4 | 0.746642781 |
| CHMP3 | 1.414419361 |  | NCAM1 | 0.746777334 |
| ATP1A2 | 1.414370509 |  | DCN | 0.755288511 |
| HERPUD1 | 1.412675262 |  | QSOX1 | 0.757201947 |
| RPS29 | 1.410053547 |  | NT5E | 0.75791079 |
| HN1L | 1.393487136 |  | DPT | 0.760383919 |
| CD1E | 1.388714579 |  | IGKV1D-33 | 0.761322679 |
| FECH | 1.388088937 |  | CLEC3B | 0.76551367 |
| DBI | 1.3875117 |  | ACTN2 | 0.769983806 |
| BECN1 | 1.38205962 |  | SERPINF1 | 0.770731362 |
| PAOX | 1.382021392 |  | TECPR2 | 0.771415486 |
| SAFB | 1.373636238 |  | VWF | 0.772710593 |
| ARIH1 | 1.371448084 |  | LAMA4 | 0.773943441 |
| RSRC2 | 1.368352363 |  | TGFB1I1 | 0.7771311 |
| SCD5 | 1.36509355 |  | NRIP2 | 0.778182271 |
| HCLS1 | 1.362352234 |  | DPYSL3 | 0.778354954 |
| RAB25 | 1.361927684 |  | PROCR | 0.779369865 |
| SQSTM1 | 1.353814447 |  | IGHV3-13 | 0.782661241 |
| AGPAT4 | 1.35302653 |  | C1orf123 | 0.78618999 |
| ABHD4 | 1.352904506 |  | PIP5K1C | 0.786969781 |
| ATOX1 | 1.348167782 |  | ILKAP | 0.787744649 |
| ARFGAP3 | 1.347719366 |  | IGHV3-64D | 0.78802866 |
| COX17 | 1.339384338 |  | OSCP1 | 0.792619349 |
| PPP1R9A | 1.337603 |  | C7 | 0.79504035 |
| TSSC4 | 1.334796637 |  | PDCD4 | 0.795812298 |
| RRP1 | 1.333723745 |  | VWA1 | 0.795977762 |
| SLTM | 1.333575858 |  | IGKV2D-24 | 0.802993462 |
| C11orf58 | 1.326900446 |  | RRAS | 0.807033219 |
| RBM19 | 1.318959308 |  | BORCS5 | 0.807973589 |
| TIMM10 | 1.313303266 |  | XPOT | 0.811706416 |
| SLC27A1 | 1.310084593 |  | MTMR1 | 0.817533566 |
| PKP2 | 1.308215257 |  | DERL2 | 0.819116127 |
| RPS21 | 1.306403073 |  | HABP2 | 0.819326243 |
| RABEPK | 1.30475597 |  | NPL | 0.820002445 |
| UBXN7 | 1.300710565 |  | PLSCR3 | 0.821208226 |
| NEK7 | 1.299259922 |  | LGALS3 | 0.822336141 |
| RPS28 | 1.290115299 |  | ELMOD2 | 0.824447994 |
| BTC | 1.287596099 |  | GNB1 | 0.825305437 |
| CASP7 | 1.282874415 |  | PCBP3 | 0.825637305 |
| GNPNAT1 | 1.282794385 |  | ITGB5 | 0.827780463 |
| BTAF1 | 1.282189922 |  | STAT2 | 0.827889516 |
| ATPIF1 | 1.273059694 |  | MGST2 | 0.829141356 |
| PTER | 1.272265713 |  | CAPN7 | 0.830953685 |
| C7orf50 | 1.271604506 |  | RAP2B | 0.83161057 |
| PTPN18 | 1.270468002 |  |  |  |
| LARP4 | 1.270106876 |  |  |  |
| TIMM8A | 1.269948466 |  |  |  |
| STMN1 | 1.269640335 |  |  |  |
| FLAD1 | 1.268971816 |  |  |  |
| SEC11C | 1.268409107 |  |  |  |
| HABP4 | 1.265668955 |  |  |  |
| SSSCA1 | 1.261569808 |  |  |  |
| FAM83H | 1.259734643 |  |  |  |
| SLC35A3 | 1.254976113 |  |  |  |
| DDX21 | 1.254662984 |  |  |  |
| BAD | 1.251553591 |  |  |  |
| CLCNKA | 1.24988039 |  |  |  |
| HMGN2 | 1.248330362 |  |  |  |
| RPL13 | 1.248244133 |  |  |  |
| L1CAM | 1.247724895 |  |  |  |
| SNRPA | 1.24698149 |  |  |  |
| COX5B | 1.244787974 |  |  |  |
| CSTB | 1.241943934 |  |  |  |
| SNW1 | 1.238522336 |  |  |  |
| ARFIP1 | 1.233279554 |  |  |  |
| COMTD1 | 1.233194135 |  |  |  |
| CCS | 1.233023111 |  |  |  |
| CBX3 | 1.230120642 |  |  |  |
| VPS37B | 1.229524084 |  |  |  |
| LNPK | 1.227871786 |  |  |  |
| RPL4 | 1.225694896 |  |  |  |
| FBXW8 | 1.224124211 |  |  |  |
| TTC19 | 1.22392064 |  |  |  |
| ATP5J | 1.222555538 |  |  |  |
| AP1M2 | 1.222182834 |  |  |  |
| NCBP3 | 1.22203025 |  |  |  |
| RANBP1 | 1.219762293 |  |  |  |
| GTF2A1 | 1.219415656 |  |  |  |
| CADM1 | 1.218156708 |  |  |  |
| PTRHD1 | 1.217903559 |  |  |  |
| LRRC59 | 1.217895257 |  |  |  |
| U2SURP | 1.21759968 |  |  |  |
| HMGB2 | 1.217481595 |  |  |  |
| TMEM141 | 1.21743107 |  |  |  |
| ASRGL1 | 1.216975406 |  |  |  |
| BRD8 | 1.216958512 |  |  |  |
| API5 | 1.215120918 |  |  |  |
| MRP63 | 1.21484328 |  |  |  |
| GOSR1 | 1.214784082 |  |  |  |
| METAP1D | 1.214758837 |  |  |  |
| RPL37A | 1.21385833 |  |  |  |
| POFUT2 | 1.213563808 |  |  |  |
| ISCA2 | 1.211588596 |  |  |  |
| RPS19 | 1.210749064 |  |  |  |
| SLC30A1 | 1.20814206 |  |  |  |
| RABL6 | 1.206727598 |  |  |  |
| MRPS18A | 1.206167275 |  |  |  |
| BCAS2 | 1.205047485 |  |  |  |
| LSM7 | 1.204538129 |  |  |  |
| NAMPT | 1.203052807 |  |  |  |
| TPD52L1 | 1.201694472 |  |  |  |
| PLGRKT | 1.201594386 |  |  |  |
| BZW2 | 1.201169685 |  |  |  |
| COX5A | 1.201053367 |  |  |  |
| PIK3CB | 1.200678562 |  |  |  |
| SF3B5 | 1.200503838 |  |  |  |
