# Supplementary material for: Proteomic analysis of human kidney biopsies unveils emerging acute kidney injury very early after liver graft reperfusion
Source: J Transl Med. 2025 Jun 16;23:658. doi: 10.1186/s12967-025-06695-w (PMC12172208; doi:10.1186/s12967-025-06695-w)
Supplement: Supplementary file 5 — Suplementary material 5. Table 5. Material used for proteomics and transcriptomics validation. For each dataset used in the in silico validation, we report the article reference, analysis type, the source of material and species, and the dataset used for comparison [file 12967_2025_6695_MOESM5_ESM.docx]

**Supplementary Table 5.** Comparative pathway analysis for pathway validation, using already published proteomic and transcriptomic datasets.

Significance threshold was set at q<0.05

| PATHWAYS | Direction of regulation | (P) Daniels et al (human serum) (26) | (P) Huang et al (rats kidney) (37) | (P) Lin et al (mouse kidney) (30) | (P) Malagrino et al (cortex) (33) | (P) Malagrino et al (urine) (33) | (P) Paranjpe et al (urine) (35) | (T) Correa-Costa et al (mouse IRI) (25) | (T) Ferreira-Malo et al (mouse IRI) (34) | (T) Gerhardt et al (mouse IRI) (28) | (T) Hinze et al (human AKI 2-3 (29) |
| --- | --- | --- | --- | --- | --- | --- | --- | --- | --- | --- | --- |
| Antigen Presentation: Folding, assembly and peptide loading of class I MHC | ↑ |  |  |  |  |  |  |  |  |  | x |
| Activation of Matrix Metalloproteinases | ↑ |  |  | x |  |  |  |  |  |  |  |
| Antigen processing-Cross presentation | ↑ |  |  |  | x |  |  |  |  |  | x |
| Assembly of collagen fibrils and other multimeric structures | ↑ |  |  |  |  |  |  |  | x |  |  |
| Class I MHC mediated antigen processing & presentation | ↑ |  |  |  | x |  |  |  |  |  | x |
| Collagen biosynthesis and modifying enzymes | ↑ |  |  |  |  |  |  |  | x |  |  |
| Collagen degradation | ↑ |  |  | x |  |  |  |  |  |  |  |
| Collagen formation | ↑ |  |  |  |  |  |  |  | x |  |  |
| Cytokine Signaling in Immune system | ↑ | x |  | x | x |  |  | x |  |  | x |
| Degradation of the extracellular matrix | ↑ |  |  | x |  |  |  |  | x |  |  |
| ECM proteoglycans | ↑ |  |  |  |  | x | x |  |  |  |  |
| Elastic fibre formation | ↑ |  |  |  |  |  |  | x |  |  |  |
| Endosomal/Vacuolar pathway | ↑ |  |  |  |  |  |  |  |  |  | x |
| ER-Phagosome pathway | ↑ |  | x |  | x |  |  |  |  |  | x |
| Extracellular matrix organization | ↑ |  |  | x |  |  |  | x | x |  |  |
| Immunoregulatory interactions between a Lymphoid and a non-Lymphoid cell | ↑ |  |  |  |  |  |  |  |  |  | x |
| Integrin cell surface interactions | ↑ |  |  |  |  |  |  | x | x |  |  |
| Interferon alpha/beta signaling | ↑ |  |  |  |  |  |  |  |  |  | x |
| Interferon gamma signaling | ↑ |  |  |  |  |  |  |  |  |  | x |
| Interferon Signaling | ↑ |  |  |  |  |  |  |  |  |  | x |
| Signaling by PDGF | ↑ |  |  |  |  |  |  | x | x |  |  |
| Complex I biogenesis | ↓ |  |  |  |  |  |  |  | x |  |  |
| Cell-Cell communication | ↓ |  |  |  |  |  |  |  |  | x |  |
| The citric acid (TCA) cycle and respiratory electron transport | ↓ |  | x | x* |  |  |  |  | x |  |  |
| Respiratory electron transport | ↓ |  |  |  |  |  |  |  | x | x |  |
| Respiratory electron transport, ATP synthesis, heat production by uncoupling proteins. | ↓ |  | x | x* |  |  |  |  | x | x |  |
| Iron uptake and transport | ↓ | x* |  |  |  |  |  |  |  | x |  |
| trans-Golgi Network Vesicle Budding | ↓ | x* |  |  |  |  |  |  |  |  |  |
| Protein localization | ↓ |  |  | x* |  |  |  |  |  | x |  |
| Metabolism | ↓ |  |  | x* |  |  |  |  | x |  | x* |
| Mitochondrial biogenesis | ↓ |  |  |  |  |  |  |  |  | x |  |
| Nephrin family interactions | ↓ |  |  |  |  |  |  |  |  | x |  |
| Neutrophil degranulation * | ↓ | x |  |  |  |  |  |  |  |  |  |
| Reversible hydration of carbon dioxide * | ↓ | x* |  |  |  |  |  |  |  |  |  |
| Clathrin-mediated endocytosis * | ↓ | x* |  |  |  |  |  |  |  |  |  |
| Cellular response to chemical stress * | ↓ | x* |  | x* |  |  |  |  |  |  |  |
| XBP1(S) activates chaperone genes * | ↓ |  |  |  |  |  | x* |  |  |  |  |
| * p<0.05 threshold was used instead of q<0.05 for the analysis, there were no or only a few pathways that passed FDR check.  Numbers within brackets: references, see below  FDR: false discovery rate; P: proteomic analyses; T: transcriptomic analyses | | | | | | | | | | | |
